# As in Real Estate, Location Matters: Cellular Expression of Complement Varies Between Macular and Peripheral Regions of the Retina and Supporting Tissues

**DOI:** 10.3389/fimmu.2022.895519

**Published:** 2022-06-15

**Authors:** Randy Zauhar, Josef Biber, Yassin Jabri, Mijin Kim, Jian Hu, Lew Kaplan, Anna M. Pfaller, Nicole Schäfer, Volker Enzmann, Ursula Schlötzer-Schrehardt, Tobias Straub, Stefanie M. Hauck, Paul D. Gamlin, Michael B. McFerrin, Jeffrey Messinger, Christianne E. Strang, Christine A. Curcio, Nicholas Dana, Diana Pauly, Antje Grosche, Mingyao Li, Dwight Stambolian

**Affiliations:** ^1^ Department of Chemistry and Biochemistry, The University of the Sciences in Philadelphia, Philadelphia, PA, United States; ^2^ Department of Physiological Genomics, Ludwig-Maximilians-Universität München, Planegg-Martinsried, Germany; ^3^ Department of Ophthalmology, University Hospital Regensburg, Regensburg, Germany; ^4^ Department of Ophthalmology, Perelman School of Medicine, University of Pennsylvania, Philadelphia, PA, United States; ^5^ Department of Biostatistics, Epidemiology and Informatics, University of Pennsylvania Perelman School of Medicine, Philadelphia, PA, United States; ^6^ Department of Orthopaedic Surgery, Experimental Orthopaedics, Centre for Medical Biotechnology (ZMB), University of Regensburg, Regensburg, Germany; ^7^ Department of Ophthalmology, Inselspital, Bern University Hospital, University of Bern, Bern, Switzerland; ^8^ Department of BioMedical Research, University of Bern, Bern, Switzerland; ^9^ Department of Ophthalmology, Friedrich-Alexander-Universität Erlangen-Nürnberg, Erlangen, Germany; ^10^ Bioinformatics Unit, Biomedical Center, Ludwig-Maximilians-University Munich, Planegg-Martinsried, Germany; ^11^ Metabolomics and Proteomics Core and Research Unit Protein Science, Helmholtz-Zentrum München, Neuherberg, Germany; ^12^ Department of Ophthalmology and Visual Sciences, University of Alabama at Birmingham, Birmingham, AL, United States; ^13^ Department of Psychology, University of Alabama at Birmingham, Birmingham, AL, United States; ^14^ Experimental Ophthalmology, University of Marburg, Marburg, Germany

**Keywords:** single cell, complement, age-related macular degeneration, retina, RPE/choroid

## Abstract

The cellular events that dictate the initiation of the complement pathway in ocular degeneration, such as age-related macular degeneration (AMD), is poorly understood. Using gene expression analysis (single cell and bulk), mass spectrometry, and immunohistochemistry, we dissected the role of multiple retinal and choroidal cell types in determining the complement homeostasis. Our scRNA-seq data show that the cellular response to early AMD is more robust in the choroid, particularly in fibroblasts, pericytes and endothelial cells. In late AMD, complement changes were more prominent in the retina especially with the expression of the classical pathway initiators. Notably, we found a spatial preference for these differences. Overall, this study provides insights into the heterogeneity of cellular responses for complement expression and the cooperation of neighboring cells to complete the pathway in healthy and AMD eyes. Further, our findings provide new cellular targets for therapies directed at complement.

## Introduction

Complement, a part of innate immunity serves as the first line of defense against foreign pathogens and altered cells. Depending on context, it is initiated by three distinct pathways: classical (CP), lectin (LP) and alternative pathway (AP). There are 40 - 60 complement proteins with various functions such as chemoattraction of immune cells, activation of leukocytes, opsonization of invading pathogens, lysis of susceptible pathogens, and synaptic pruning (1–4). The effector functions of the complement system are controlled through proteolytic generation of activation fragments that either bind to cell receptors or covalently attach to cell surfaces adjacent to sites of complement activation. It is the job of membrane bound regulatory molecules to modulate complement pathway activation proportionally to limit damage to host tissues (5, 6). The primary site of biosynthesis for the majority of the fluid-phase complement proteins is the hepatocyte and more than 90% of plasma complement is derived from the liver. Extrahepatic cells such as macrophages, endothelial cells and neurons can also produce complement constitutively and when induced (7).

CP activation is dependent on the binding of the recognition molecule C1q to patterns like IgM and IgG immune complexes, RNA, DNA, phosphatidylserine, CRP and others while the LP is activated when mannose binding lectin (MBL) or ficolins (FCN) bind to carbohydrate structures. Following activation, the CP and LP lead to successive cleavage of C4 and C2 and formation of the C3 convertase [C4bC2bC3b]. The AP is activated by spontaneous hydrolysis of C3 to C3(H_2_O) that subsequently binds complement factor B (FB). Cleavage of FB to Bb and Ba by complement factor D (FD) leads to formation of the AP C3 convertase [C3b(H_2_O)Bb]. Of note, the AP includes an amplification loop for the CP and LP through the action of C3(H_2_O)Bb on C3 to generate C3b which forms C3bBb and additional cleaving of C3. Finally, generation of C3b by any of the three pathways will lead to the generation of the C5 convertase and the common terminal pathway (1–4).

Structures such as the brain and eye have their own local complement expression due to the inability of bloodborne complement proteins to pass through the blood-brain and blood-retinal barriers (8–12). The retina is a specialized light-sensitive multilayered tissue composed of neurons, glia, and vasculature. The retinal pigment epithelium (RPE) is a monolayer of pigmented cells that metabolically supports outer retina and participates in the renewal of photoreceptor outer segments (13, 14). The underlying choroid is a specialized component of the systemic circulation, with pericytes/smooth muscle cells, fibroblasts, melanocytes, neurons, and immune cells (15). Understandably, it is important to determine the complement expression of the distinct cell types in the human retina, RPE and choroid. The idea of a local complement biosynthesis in the retina, RPE and choroid has been supported by earlier studies (16, 17). Importantly, several eye diseases such as glaucoma, diabetic retinopathy, autoimmune uveitis and age-related macular degeneration (AMD), have reported genetic associations with complement (18–20). A large effort of AMD research has been complement based due to strong evidence for complement dysregulation and the identification of complement protein in extracellular deposits called drusen (21, 22). C3, complement receptor 1 (CR1), and terminal complement proteins C5b-9 (membrane attack complex, MAC) have been identified within drusen (23–25). In addition, genome wide association studies (GWAS) have reported significant associations of *complement factor H (CFH)*, *C3*, *complement factor I (CFI)*, *complement factor 9 (C9)*, and *complement factor B (CFB)* with AMD, further evidence that complement is involved in AMD (26).

AMD is a major cause of visual impairment in patients over the age of 65 (27). Currently it affects about 200 million worldwide and is predicted to increase to 300 million by 2040 (28). For reasons still being learned, AMD primarily affects the macula region (29), a specialized region in the retina of humans and non-human primates. In its advanced stages, there are two forms of AMD, geographic atrophy (GA; dry AMD) and choroidal neovascularization (CNV; wet AMD) (30). Both forms are associated with degeneration of photoreceptors, RPE and choroid. Although the current treatment for the wet form of AMD are intraocular injections of antibodies directed at vascular endothelial growth factor (VEGF) many of these treated patients go on to develop atrophy and further vision loss (31–33). Currently, there is no effective treatment for dry AMD. Biochemical, histological, and genetic studies have implicated several pathways involved in AMD, including oxidative damage, chronic inflammation, complement system malfunction and dysregulation of lipids as well as extracellular matrix (34, 35).

Recent clinical trials have utilized drugs to delay AMD progression through alteration of the complement pathway (reviewed in [36–38)]. C3 inhibitors have received the most attention due to the dominant role of C3 as a control point for all three complement pathways. POT-4, a compstatin derivative and C3 inhibitor was used in clinical trials but had to be terminated due to lack of efficacy (39, 40). A second derivative of POT-4, APL-2 (Pegcetacoplan*)*, recently completed phase 3 trials and showed a decrease in GA growth by 25% but was complicated by new onset choroidal neovascularization (CNV) (41). Clinical trials aiming to inhibit C5 have been met with modest success. Avacincaptad Pegol, a C5 inhibitor, was effective at reducing geographic atrophy (GA) growth by 28% but also suffered from a higher onset of CNV in the treated group (41, 42). Monoclonal antibodies directed at C5, Eculizumab and LFG316, have been used to treat GA but were unsuccessful in reducing GA progression (43). Other clinical trials directed at complement factors FD, properdin and FB did not show clinical efficacy (43, 44). Recent reports have raised concerns about treating GA with complement inhibitors (44). Reasons for these failures or modest successes with complications might include route of administration, inappropriate target cells for modulation, failure to select patients most likely to benefit, insensitive trial endpoints, and limited understanding of both complement expression in healthy retina, RPE and choroid and the role of complement in AMD pathophysiology.

This urgent need to assess the complement gene expression in single cells, resolve cell types, characterize the signature of complement expression across cells, and identify differences in health and disease can now be met implementing recent technological breakthroughs in single cell RNA-sequencing (scRNA-seq) (45–47). To the best of our knowledge, this is the first comprehensive study that describes the single cell RNA and protein expression of complement in the human retina, RPE and choroid. Protein expression was determined for many of the complement expressing genes either by mass spectrometry or western blotting. We also compared our human complement expression to our previously published mouse complement single cell retina data to report differences that should be considered before testing drugs targeted for humans in preclinical mouse studies (48). Finally, we identified local complement expression changes in retina and choroid from post-mortem eyes affected with early AMD. Our results underscore the power of single cell technologies to gain deeper insight into complement homeostasis and assist in our understanding of the complement dysregulation occurring in AMD. Our results increase the knowledge base that exists for ocular complement and will provide investigators with essential additional information to design novel therapies.

## Results

### Atlas of Complement Expression in the Human Retina, RPE and Choroid Identified by scRNA-seq

Aiming to better understand the contribution of locally produced complement components to immune homeostasis in the posterior part of the human eye, we generated complement expression profiles for all cell types of the human retina, RPE and choroid *via* scRNA-seq (**Table S1**). Cell clusters were annotated using known gene markers for retina and choroidal cell types and clusters sharing the same markers were combined into 11 cell types for the retina and 10 cell types for the choroid (49–51). To analyze the RPE for complement expression we re-processed the scRNA-seq data from Voigt et al (51).

**Figure 1:**
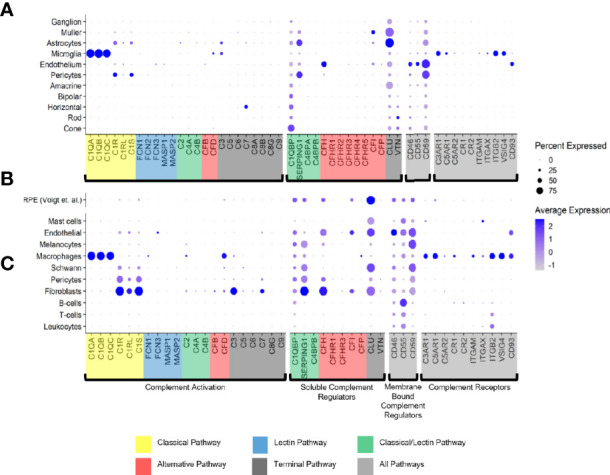
Cell type specific complement expression in the combined macula and peripheral human retina, choroid and RPE. **(A)** Dot plot showing expression pattern of genes of the complement pathways across cell types identified in the combined macula and peripheral retina. The dot plot was generated using Dotplot in the R Seurat package. The size of the dots represents the percentage of cells that expressed gene markers while color shows average expression levels of gene markers. **(B)** Reprocessed combined macula and peripheral RPE data from Voigt et al. (51). **(C)** Dot plot showing expression pattern of known gene markers across cell types identified in the combined macula and peripheral choroid.

**Figure 2:**
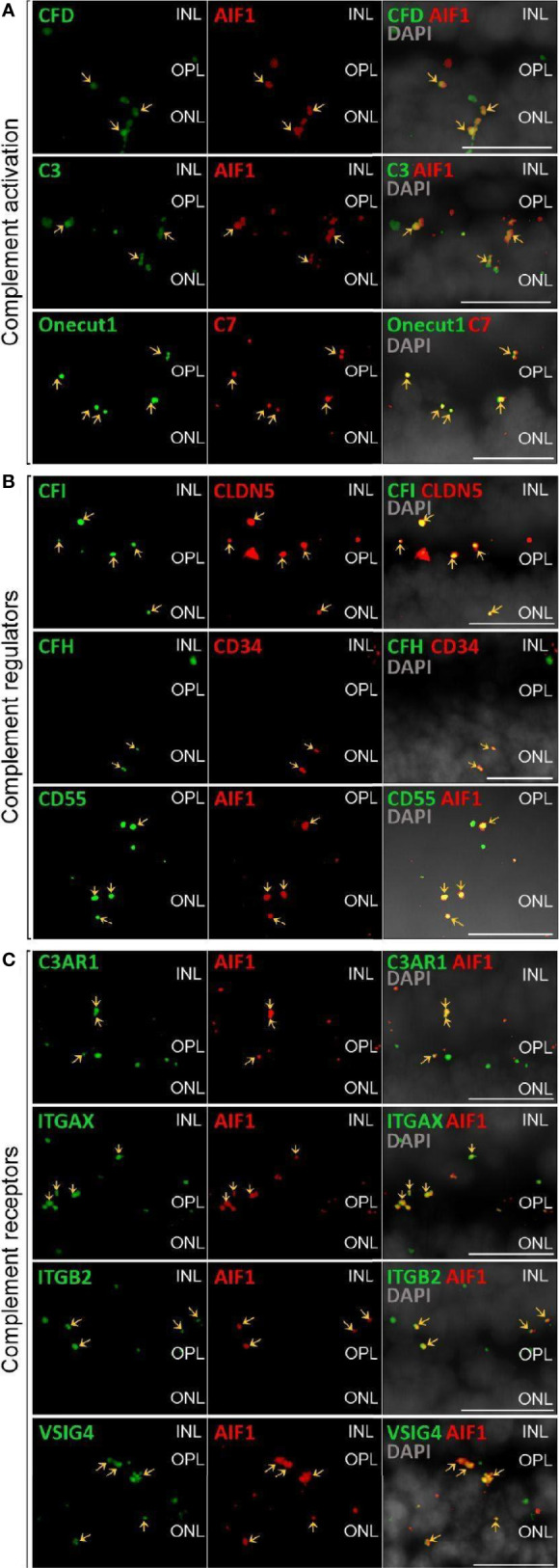
Validation of scRNA-seq for retinal complement component expression by *in situ* hybridization. **(A)** Factors driving complement activation were chosen for detection by *in situ* hybridization. *CFD* expression from microglia was detected in the inner nuclear layer (INL), outer plexiform layer (OPL) and outer nuclear layer (ONL), but was mostly associated with microglia expressing *AIF1* (alias *IBA1*) in the OPL. *C3* expression was mostly limited to microglia located in OPL. *C7* transcripts were detected in the OPL with overlap of signal with the horizontal marker Onecut1 in the OPL. **(B)** Soluble (*CFH, CFI*) and membrane-bound (*CD55*) complement regulators primarily colocalized with vascular cells and microglia. Signals of *CFH* transcript were strongest from INL and OPL and overlapped with the endothelial marker *CD34*. *CFI* expression associated with the endothelial marker *CLDN5* was strongest in the INL and OPL. *CD55* was robustly expressed in microglia expressing *AIF*. **(C)** Complement receptor *C3AR1* transcripts were detected at low levels in the INL and OPL and co-localized with the microglia marker *AIF1*. *ITGAX* was robustly expressed in the OPL and co-localized with the microglia marker *AIF1* in the OPL. *ITGB2* transcripts were less abundantand co-localized with *AIF1* in the OPL. Also *VSIG4* was strongly expressed in OPL and ONL co-localizing with the microglia marker *AIF1*. **(A–C)** Arrows indicate co-localization of the gene of interest with respective cell marker. Scale bars, 20 µm.

Only a few complement activators were expressed in the retina and included *C1QA-C*, *C1R, C1RL, C1S, CFB, CFD, C3* and *C7* (**Figure 1A**). Activators for the LP, *FCN1/3* and *MASP1/3*, had minimal expression. By *in situ* hybridization, *CFD* and *C3* transcripts were confirmed in microglia (**Figure 2A**). Moreover, the *C7* expression that surprisingly was confined to horizontal cells was verified as well (**Figure 2A**). Of the cells expressing secreted complement components, microglia had the highest transcription especially for *C1QA-C*. Surprisingly, the RPE had negligible expression of complement activators (**Figure 1B**), while all the activators for the CP were expressed in the choroid, albeit not in every cell type (**Figure 1C**). In the choroid, macrophages had the highest expression of *C1QA-C* while fibroblasts demonstrated robust expression signals for *C1R, C1RL* and *C1S*. Of note, *C3* the central component of the complement pathway was robustly transcribed in fibroblasts (**Figure 1C**).

The soluble complement regulators *component 1q subcomponent binding protein* (*C1QBP*) and *clusterin* (*CLU*) were transcribed by all the retinal cell types, while *CFH* transcription was mostly confined to the endothelium (**Figures 1A**, **2B**). A modest *CFI* expression signal was present in Müller glia and endothelium (**Figures 1A**, **2B**). SERPING1, a regulator dissociating the C1 complex by binding C1r and C1s, showed robust expression in both pericytes and astrocytes. In the RPE, we found moderate expression of regulators *C1QBP*, *SERPING1*, *CFH*, *CFI* and robust expression of *CLU* (**Figure 1B**). Finally, most of the regulators were expressed in various cell types of the choroid (**Figure 1C**). Choroidal fibroblasts had robust signals for *SERPING1*, *CFH* and *CLU*. It should be noted that *CFHR2* and *CFHR5* had no detectable expression in the retina and RPE/choroid. *CFHR1*, *CFHR3* and *CFHR4* expression was weak and limited to specific cell types in the retina and RPE/choroid.

The membrane bound complement regulators *CD46*, *CD55* and *CD59*, demonstrated robust expression in retinal vascular endothelium (**Figure 1A**). Surprisingly, *CD55* transcripts were also found by *in situ* hybridization to co-localize with the microglia marker *AIF1* (**Figure 2B**). CD59, which blocks membrane perforation of C5b-9, is expressed on all retinal cell types to varying degrees. Positive expression of *CD59* across all retinal cell types would make the retina resistant to MAC damage unless the regulators became overwhelmed. A similar conclusion would apply for the RPE and choroid due to moderate to robust expression of all three membrane bound regulators in most cell types. Noteworthy is the absence of *CD59* from choroidal B and T cells.

Finally, we identified the cell types expressing complement receptors which would make these cells most responsive to changes in local complement homeostasis. The integrin family receptors, *ITGAM*, *ITGB2G* and *ITGAX*, bind iC3b facilitating immune clearance and phagocytosis (1). Retinal microglia expressed all three receptors (**Figures 1A**, **2C**). *VSIG4*, an immunoglobulin superfamily receptor, is also highly expressed in retinal microglia and is responsible for phagocytosis of C3b and iC3b (1). *CD93*, involved in clearance of apoptotic cells, is expressed in retinal endothelial cells. *C3AR* and *C5AR*, receptors for C3a and C5a, are modestly expressed in microglia cells. This microglia-specific expression pattern could also be confirmed by *in situ* hybridization for *C3AR*, *ITGAX*, *ITGB2* and *VSIG4* (**Figure 2C**). Interestingly, except for *C5AR1*, none of the complement receptors were detectable by scRNA-seq in the RPE. Choroidal macrophages demonstrated a similar expression profile to retinal microglia (**Figure 1**). In addition, choroidal T cells and leukocytes expressed *ITGB2*.

### Differential Expression Between Normal Macula and Peripheral Retina and Choroid

Significant differences in complement expression were detected in healthy retinal cells from the macula and peripheral retina (**Data File S1**). We found transcript reduction in the macula for *CFI* in astrocytes, *CLU* in bipolar, horizontal and ganglion cells as well as *VTN* in rods. In contrast, *VTN* in cones, *CD46* in endothelial cells and *CD59* in pericytes were increased in the macula compared to the periphery.

Similarly, a number of choroidal genes showed significant expression differences between cells located in sub-macula and peripheral regions (**Data File S1**). These differences included decreased *CLU* in Schwann cells, *C3* in macrophages, *CFI* and *C1R* in endothelial cells, *CFH* in pericytes, *C1R*, *C1S*, *CLU*, *CFD*, *C3*, *CD55* and *SERPING* in fibroblasts, and *CFH* and *SERPING* in melanocytes. We found increased expression in the sub-macula region compared to the periphery in macrophages for *C1Q*, *C2* and *VSIG4*.

### Complement Transcriptome of Human Retinal Cell Types, RPE and Choroid Partially Translated Into Protein Expression

**Figure 3:**
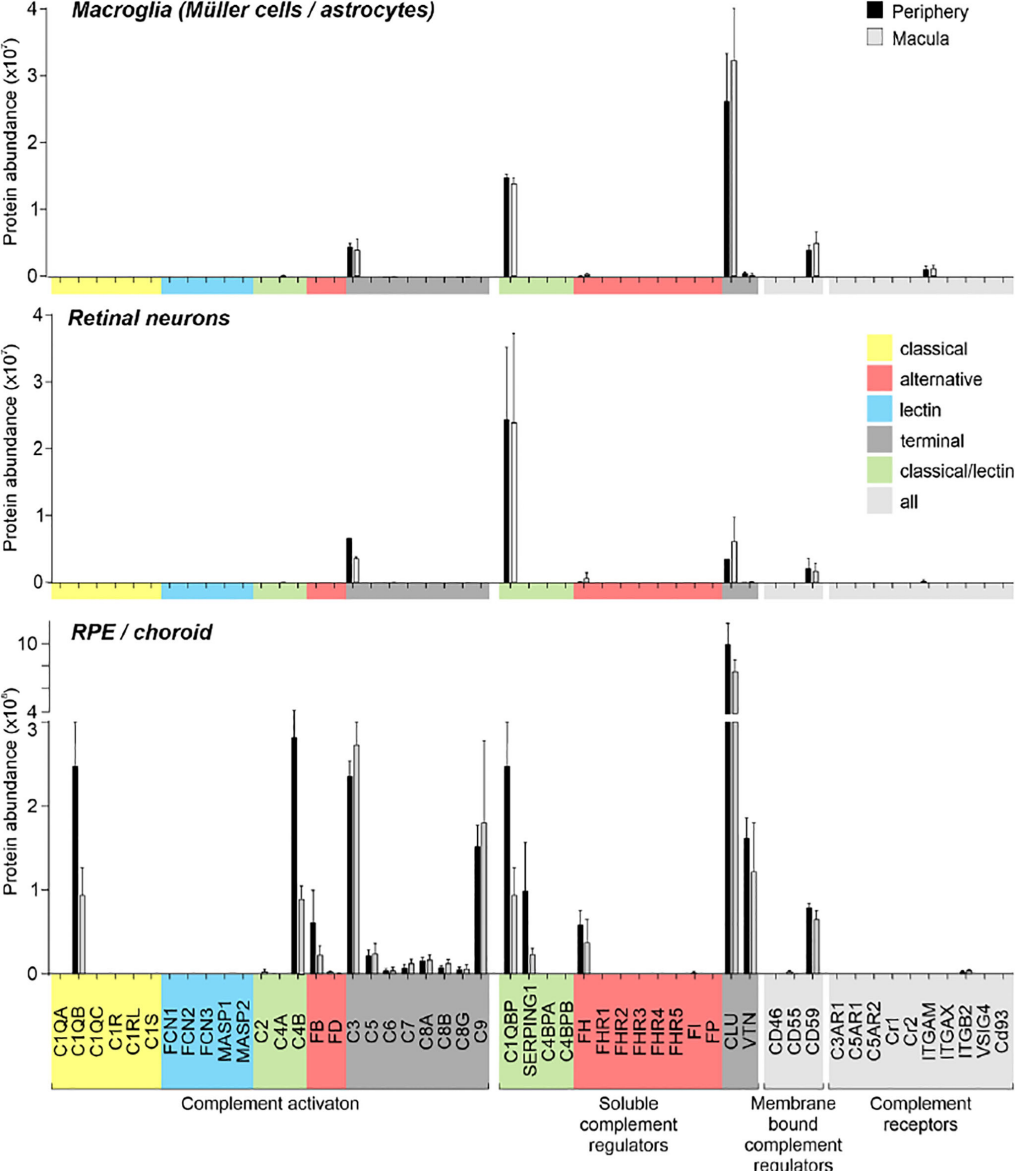
Complement proteome of macroglia (Müller cell, astrocytes), retinal neurons and RPE/choroid in the macular and peripheral human eye. Müller cells and retinal neurons were purified from peripheral and macular retinal tissue punches (6 mm in diameter) from 5 donor retinae (**Table S1**) by magnetic-activated cell sorting (MACS) and were submitted to quantitative LC MS/MS mass spectrometry. Contamination with astrocytes is likely, because no surface marker yet separates them clearly from Müller cells. Müller cells definitely outnumber astrocytes. Macroglia and neurons were depleted from ITGAM (alias CD11B)-positive microglia/macrophages and CD31-positive vascular cells. RPE/choroid was collected after the removal of retinal tissue and comprised a mixture of RPE and choroidal cell types including pericytes, endothelial cells, fibroblast and immune cells. Given the intense perfusion of choroidal tissues, these samples do not allow unequivocal discrimination of the source especially of soluble complement components as they could be expressed by local cell types or by liver cells and enter the choroid *via* the circulation.

**Figure 4:**
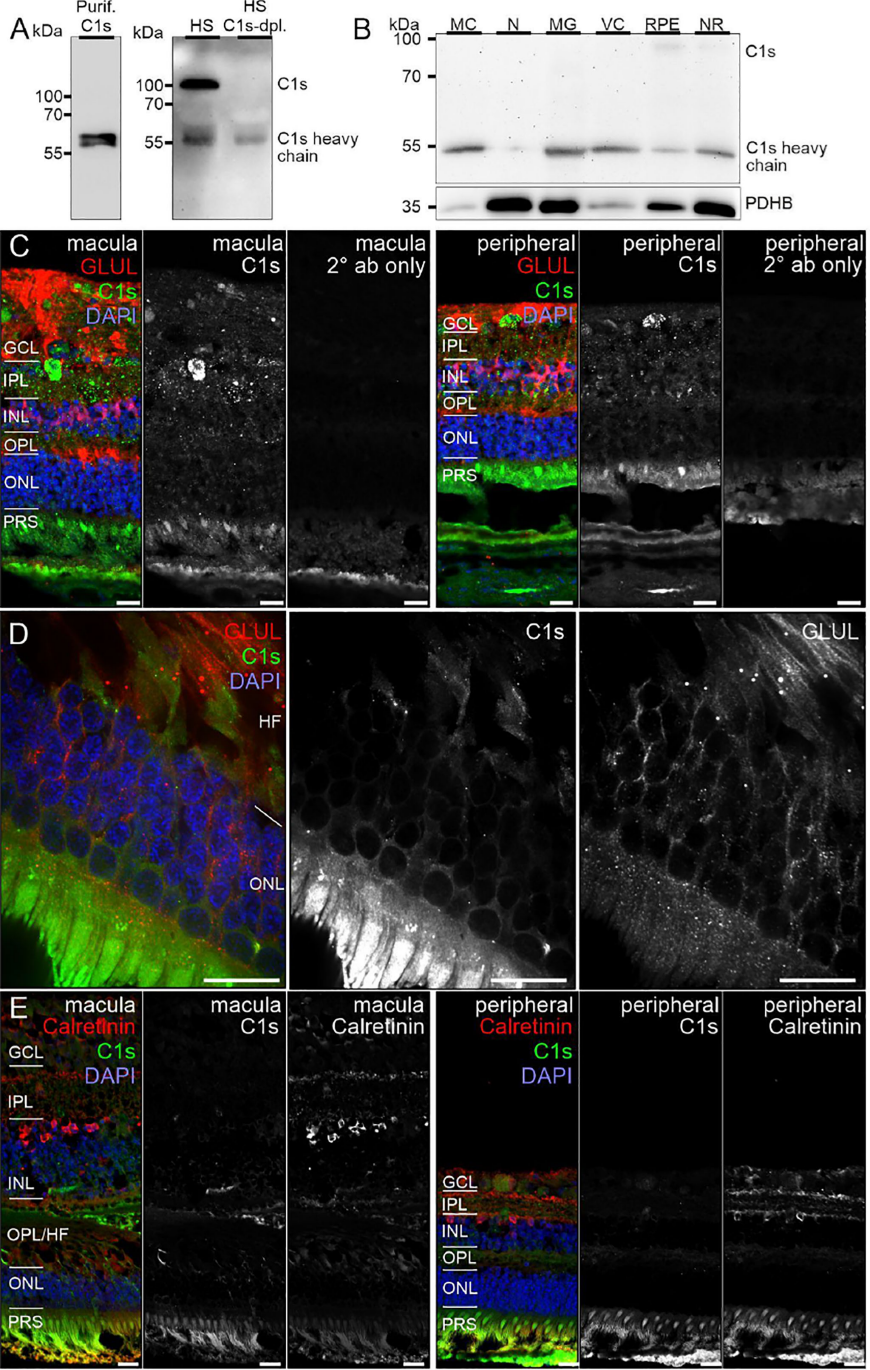
C1s protein expression in the human retina. **(A)** Full C1s (~75 kDa) and the C1s heavy chain (~47 kDa) were reliably detected by Western blot using purified C1s protein and human serum (HS). C1s-depleted HS still contained remaining C1s heavy chain, while full length C1s was absent. **(B)** Western blot analysis of C1s on purified retinal cell types from peripheral human retina and RPE/choroid. The total protein extracted per cell population was loaded. PDHB (pyruvate dehydrogenase beta subunit) served as housekeeper that has been shown to be expressed at equal levels in all investigated cell types. In contrast to HS, primarily the heavy chain of C1s was detected in retinal samples. C1s levels were highest in Müller cells (MC) and vascular cells (VC). MG, microglia; NR, whole neuroretina. RPE, retinal pigment epithelium including choroid. **(C)** Representative micrographs of C1s-stainings from macular (*left*) and peripheral (*right*) retinae. Photoreceptors and cells of the ganglion cell layer (GCL) as well as punctate structures in the inner plexiform layer (IPL) displayed highest labeling intensities. Sections were co-stained for the Müller cell marker glutamine synthetase (GLUL). **(D)** Higher magnification of the outer nuclear layer (ONL) and Henle fiber layer (HL) in the macular retina showed a partial overlap of C1s and GLUL. **(E)** Co-staining of C1s with calretinin, a marker of inner retinal neurons such as ganglion and amacrine cells, yielded no considerable overlap. **(C-E)** INL, inner nuclear layer; OPL, outer plexiform layer; PRS, photoreceptor inner and outer segments. Scale bars, 20 µm.

**Figure 5:**
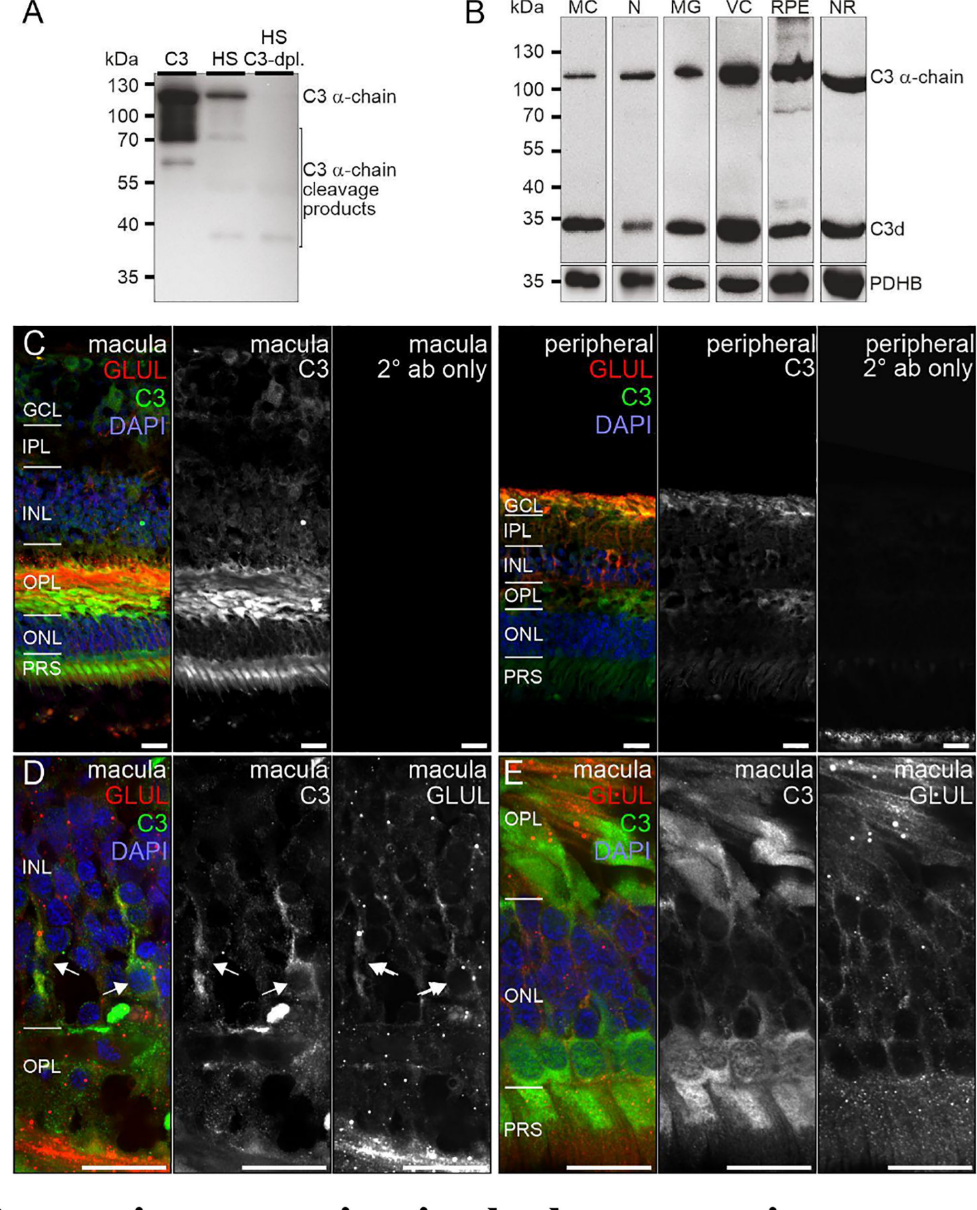
C3 protein expression in the human retina. **(A)** The C3 α-chain (~120 kDa) and its cleavage products were robustly detected by Western blot if purified C3 or human serum (HS) was loaded. No characteristic bands were detected in C3-depleted HS. **(B)** Western blot analysis of C3 on purified retinal cell types from peripheral human retina and RPE/choroid. The total protein extracted per cell population was loaded. PDHB served as housekeeper since not the exact same amount of protein could be loaded given the low protein yield from the microglial (MG) and vascular cell (VC) populations. The C3 α-chain and its cleavage product C3d were detected in every retinal cell population at comparable levels. MC, Müller cells; N, retinal neurons; NR, whole neuroretina; RPE, RPE/choroid mixed samples. **(C)** Representative micrographs of C3-stainings from macular (*left*) and peripheral (*right*) retinae. Photoreceptors, photoreceptor terminals and Müller cell processes were positively labeled. Co-staining for the Müller cell marker glutamine synthetase (GLUL) demonstrated an overlap primarily in cells from the peripheral retina. **(D)** Higher magnification of the inner nuclear layer (INL) highlighting the co-localization of C3 and GLUL (arrows). **(E)** Co-staining of C3 and GLUL did not result in an overlap in the outer retina. C3 stained cone photoreceptors (asterisk) which form a single row excluding rod photoreceptors from that layer in the central retina. **(C–E)** GCL, ganglion cell layer; IPL, inner plexiform layer; OPL, outer plexiform layer; ONL, outer nuclear layer; PRS, photoreceptor inner and outer segments. Scale bars, 20 µm.

**Figure 6:**
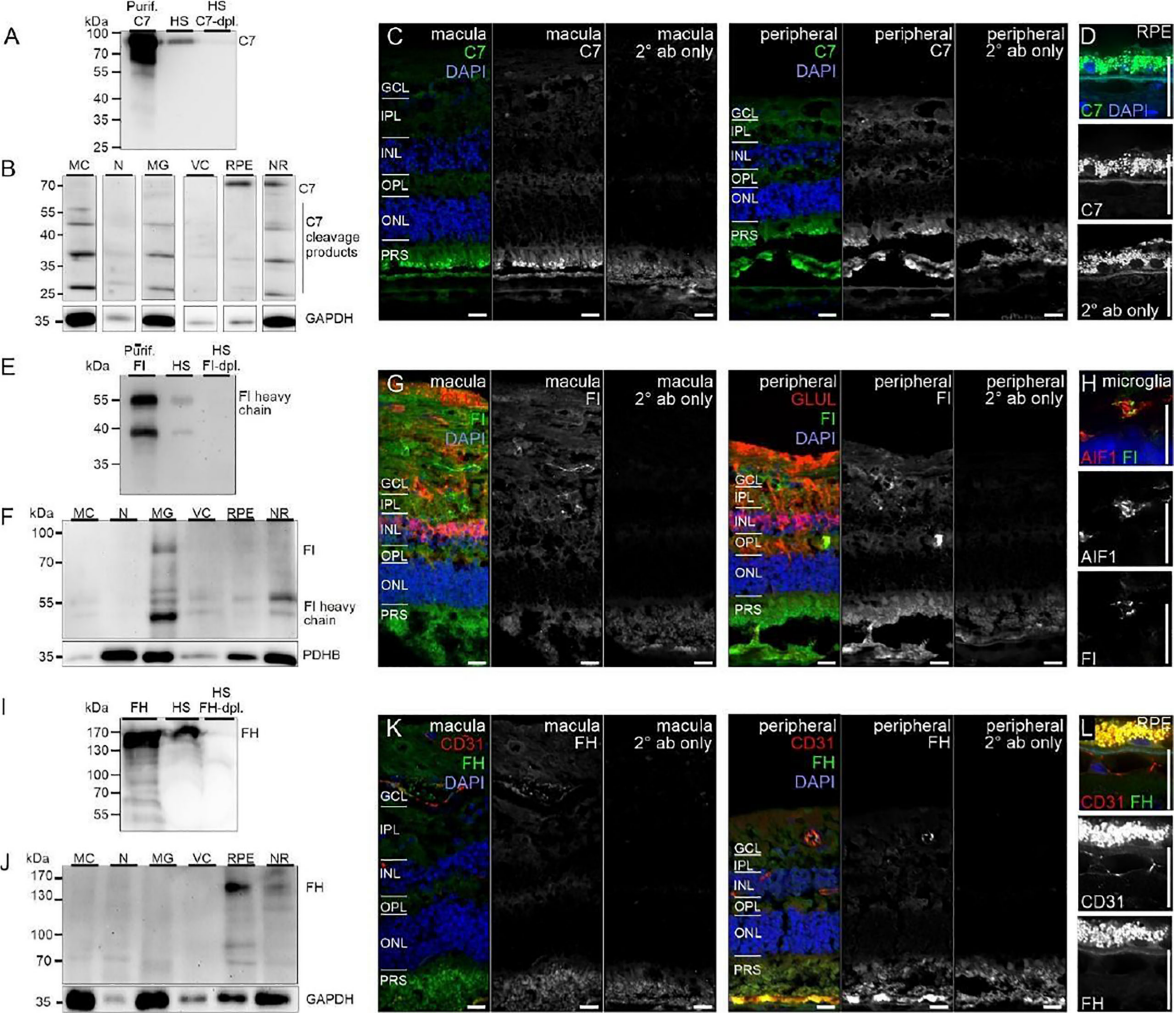
CFI, CFH and C7 protein expression in the human retina. **(A)** Loading purified C7 or human serum (HS), full length C7 was detected as a single band (~90 kDa) and cleavage products <90 kDa. **(B)** Full-length C7 was detected at low levels in purified cell types, but robustly in RPE/choroid. Cleavage products were detected in all cell populations. C7 immunoreactivity was similar in macular and peripheral retinal sections. **(D)** Higher magnification of the C7 staining in RPE. **(E)** Loading purified CFI or HS, only the CFI heavy chain (~50 kDa) was unequivocally detected by Western blot. **(F)** CFI was present in Müller cells (MC), microglia (MG), vascular cells (VC) and RPE/choroid, but not in neurons. **(G)** Comparable CFI-labeling of structures of the macular and peripheral retina. CFI-immunoreactivity partially overlapped with that of the Müller cell marker glutamine synthetase (GLUL). **(H)** Higher magnification of the inner plexiform layer (IPL) demonstrating co-localization of CFI with IBA1-positive microglia. **(I)** Detection of CFH (~150 kDa) by Western blot using purified CFH and HS. **(J)** CFH was only detected in RPE/choroid. A contamination with CFH from the system circulation cannot be excluded. **(K)** CFH immunoreactivity was confined to vessel lumens. **(L)** Minor CFH immunoreactivity at the RPE – Bruch’s membrane interface at higher magnification. **(B, F, J)** PDHB or GAPDH (Glyceraldehyde-3-phosphate dehydrogenase) served as housekeepers. N, neurons; NR, whole neuroretina. **(C, D, G, H, K, L)** GCL, ganglion cell layer; OPL, outer plexiform layer; ONL, outer nuclear layer; PRS, photoreceptor segments. Scale bars, 20 µm.

It is known that transcript expression does not necessarily correlate with protein levels (52, 53). This has also been confirmed at the single cell level under very well-controlled conditions and underscores the necessity of measuring proteins as well as RNA (54). Accordingly, we validated select transcripts at the protein level (**Figures 3**–**6**). Purified retinal cells were obtained by sequential magnetic-activated cell sorting (MACS) of ITGAM-positive microglia, CD31-positive vascular cells (endothelium, pericytes), and CD29-positive Müller glia, while the remaining cell population consisted of photoreceptors and other neuronal types (**Figure S1**) (48). The cell populations with high yield in terms of cell numbers recovered after MACS (Müller glia, neurons, RPE/choroid) were subjected to LC-MS/MS mass spectrometry (**Figure 3**). The level of cell enrichment was determined by marker gene expression and marker protein abundance (**Figure S1**).

Remarkably, consistent with scRNA-seq, the soluble complement regulators CLU and C1QBP as well as the membrane bound regulator CD59 were detected at protein level (via LC-MS/MS mass spectrometry) in all cells of the inner and outer retina and in most cell types of the RPE/choroid, albeit at varying levels and rates (**Figure 3**). Western blot analysis of some selected candidates confirmed that their identified RNAs were translated (**Figures 4**–**6**). C1s is an esterase cleaving C2/C4, thereby facilitating the formation of the CP C3-convertase. C1s peptides were not detected by LC-MS/MS mass spectrometry possibly because this method is less sensitive to proteins of low abundance (**Figure 3**). Western blot did detect C1s heavy chain in the Müller cell population, which includes astrocytes, and also in the CD31-positive vascular cell population (**Figure 4B**). Thus, the findings of *C1S* transcripts in astrocytes, Müller cells, and pericytes (**Figure 1A**) by scRNA-seq were confirmed. To a lesser extent, C1s protein was also found in microglia, RPE/choroid and retinal neurons (**Figure 4A**).

C3, the central protein of the complement system, is under tight regulation to prevent inadvertent complement activation. *C3* transcript detection using scRNA-seq was mainly restricted to microglia and astrocytes in the retina and to fibroblasts in RPE/choroid (**Figure 1**). Its protein was also detected in or on purified retinal neurons *via* LC-MS/MS mass spectrometry (**Figure 3**) and western blot (**Figure 5**). Also, C3 cleavage product, C3d, was detected in or on all purified retinal cell types (**Figure 5A**).

Also, VTN and components of the terminal pathway (C5, C6, C8A, C8B, C8G, C9) proteins were detected in RPE/choroid samples (**Figure 3**). However, protein analysis sometimes provided contradictory results. This challenge of detecting secreted proteins is clearly reflected in our data. LC-MS/MS mass spectrometry is an unbiased screening method that can miss complement components already secreted and not tightly attached to cell surfaces. As a result, lower levels of intracellular proteins might be under the detection limit. The same is true for western blot analyses of enriched retinal cell populations, although they are more sensitive than LC-MS/MS mass spectrometry. To achieve the most complete picture of protein localization in the tissue, we added immunostaining to detect protein accumulation of select complement components at protein level, irrespective of the actual, expressing source in the tissue. C1s localized to the ganglion cell layer (GCL) and at spots all over the retina possibly reflecting its secretion into the interstitial space (**Figures 4C–E**). C3 was specifically detected on cones of the macular retina (**Figure 5E**), and also Müller cells (**Figure 5D**) – the latter being in line with transcript data.

Similarly, protein expression was determined for additional complement factors. C7 stood out from the list of terminal complement components, since scRNA-seq indicated a very specific expression by horizontal cells (**Figure 1**). However, LC-MS/MS mass spectrometry detected C7 in RPE/choroid samples only (**Figure 3**), while western blot detected whole C7 in neuroretina as well as RPE/choroid and C7 cleavage products in all retinal cell populations (**Figure 6B**). In support of this finding, C7 immunoreactivity was detected across the whole retinal section (**Figures 6C, D**).

Acting together with FH, FI inactivates C3b through sequential cleavage to iC3b, C3c, C3dg and finally C3d. Western blotting identified FI in/on microglia, Müller cells and CD31-positive vascular cells, but very little association with neurons or RPE/choroid (**Figure 6F**). The location of FI in microglia was confirmed by immunostaining (**Figure 6G**). FH, an additional regulator of complement activation, inactivates C3b in the presence of FI. LC-MS/MS mass spectrometry and western blots identified FH in RPE/choroid (**Figure 3**; **Figure 6J**). Since contamination of the RPE/choroid samples with systemic FH protein is likely, these results have to be interpreted with caution. Immunostaining located FH on the luminal side of retinal and choroidal vessels (**Figures 6K, L**), consistent with our scRNA-seq result of the endothelial cell’s robust expression of *CFH* (**Figure 1**).

### Human and Murine Retinal Cells Showed Species-Specific Complement Transcriptomes

**Figure 7:**
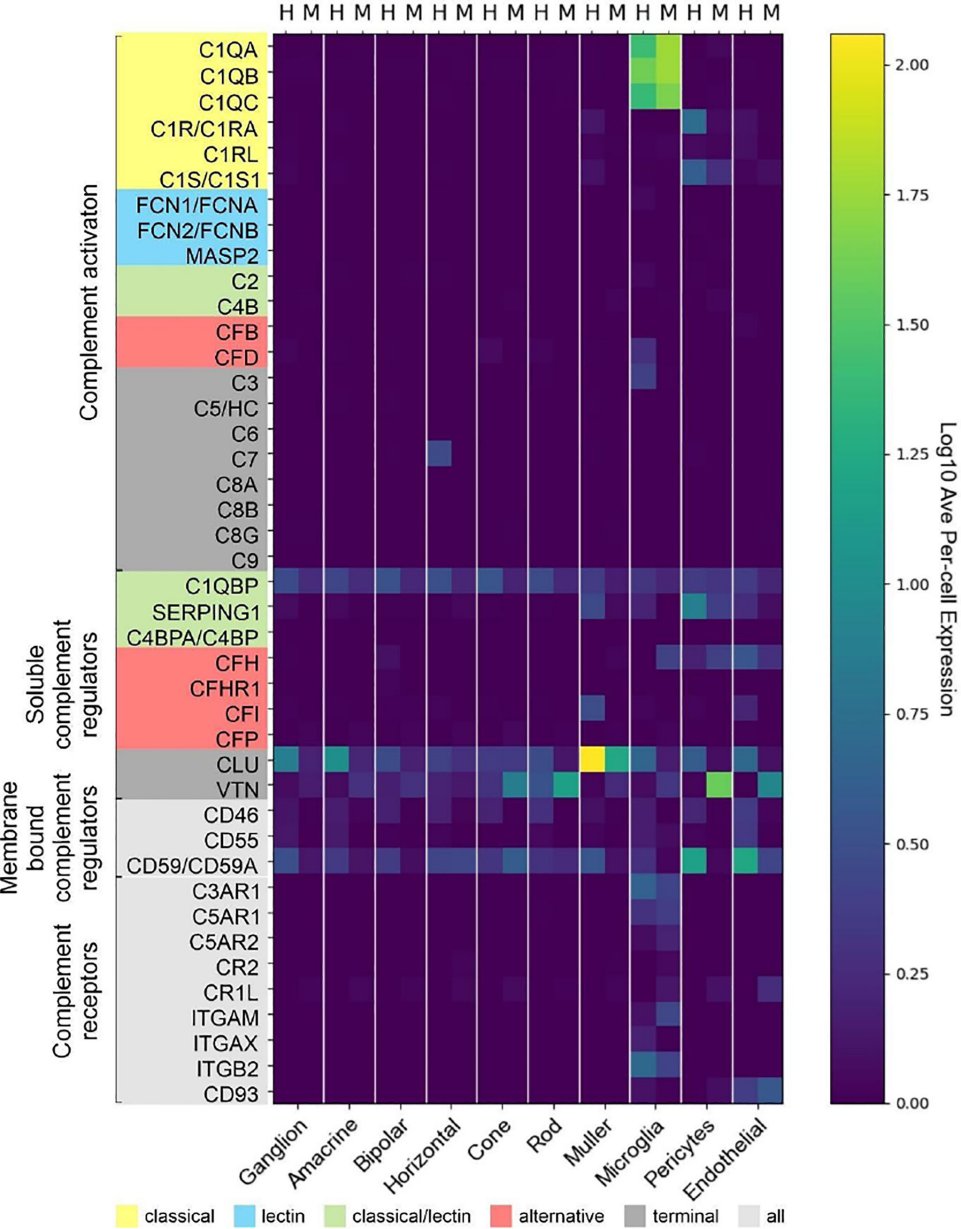
Comparison of cell type-specific complement expression between peripheral human (H) and mouse (M) retina. Expression of complement components was determined by scRNA-seq. For cross-species comparison data from the present study on human retina was compared to recently published data from mouse retina (48). Expression is measured in units of FPKM (Fragments Per Kilobase of transcript per Million mapped reads) and color-coded on a logarithmic scale.

Experimental approaches to investigate human diseases and underlying complement action involved studies in mouse models (48, 55–57). To facilitate the transfer of murine complement results to the human system, we compared the scRNA-seq expression of different complement components in the normal human peripheral (rod-dominated) retina and our own published study on rod-rich mouse retina (**Figure 7**) (48). Several differences in complement expression were observed between both species. For the CP, elevated expression levels were found in human compared to mouse retina: *C1Q*, the molecular recognition component for activation of the CP, is exclusively expressed by microglia in mice and humans, with high expression of all three components of this complex (*C1QA*, *C1QB* and *C1QC*) in both human and mouse (**Figure 7**). The remaining components of the C1 complex, *C1R* and *C1S*, which activate C4 and C2 upon recognition of antigen-antibody complexes, show significant overlapping expression in pericytes. Human pericytes and astrocyte/Müller cell fractions express both *C1R* and *C1S* at similar levels. Mouse pericytes and endothelial cells also express these transcripts, whereby *C1S* is detected at higher levels than *C1R* in the mouse. LP proteins were almost not detectable in both mouse and human, suggesting this pathway has a negligible role in maintaining cellular homeostasis in the normal eye. With respect to the AP activators, *CFD* is expressed in human microglia and not detectable in scRNA-seq, but with qPCR, in the mouse retina. *C3*, involved in both CP and AP, is especially detected in human microglia (**Figure 7**). Murine microglia showed much lower expression levels (48).

The soluble regulators of the CP demonstrated differences between human and mouse (**Figure 7**). *C1QBP* expression was more robust across all human cell types compared to mouse. *SERPING1* also showed higher expression in human than in murine endothelial cells, microglia, Müller cells and pericytes. The soluble regulators of the AP, *CFH* and *CFI*, showed very large differences. *CFH* was strongly expressed in human endothelial cells, while its expression in mouse was highest in microglia and pericytes. Interestingly, FI which acts in concert with FH, showed high expression in human endothelium and Müller glia but was almost undetectable in the mouse retina (**Figure 7**) (48). *VTN* and *CLU*, both soluble negative regulators of all complement pathways, are differentially expressed across all cell types, with expression of *CLU* higher in human than mouse in all cell types except cones. *VTN* is expressed at higher levels in mouse compared to human in all cell types except horizontal cells.

Overall, we found higher levels for the terminal soluble and membrane bound complement regulators in the human retina compared to the mouse retina (**Figure 7**). *CD46* was expressed in most human retinal cell types, but had no expression in mouse retina. Similarly, *CD55* was only expressed in human, specifically in microglia, endothelial and ganglion cells. Finally, *CD59* is expressed broadly, but differentially, across cell types, with expression generally being higher in human than in mouse in all cell types except cones. *CD59* expression is absent in mouse microglia and pericytes.

Notably, we found more agreement of complement receptors for microglia in mouse and human. While mouse microglia express all three anaphylatoxin receptors, human microglia express only *C3AR1* and *C5AR1*. The expression of the integrins *ITGAM*, *ITGAX* and *ITGB2* are also exclusive to microglia. *ITGAX* and *ITGB2* show significant expression in human microglia, *ITGAM* and *ITGB2* in mouse microglia. The complexes of these integrins, ITGAM/ITGB2 and ITGAX/ITGB2, termed CR3 and CR4 respectively, recognize inactivated complement C3 (iC3b) on cell surfaces (58–60).

We also found interesting species-specific differences with respect to proteins. C3 protein was detected *via* western blot in all purified human retinal cell types (**Figure 4B**), while it could only be identified in samples from murine RPE and neurons (48). Moreover, the complement inhibitor FH was present in samples from all mouse retinal cell types except for vascular cells (48), but on the other hand FH protein was only detectable in human RPE (**Figure 6J**).

In summary, there are distinct complement expression differences between the mouse and human retina which should be considered when using the mouse as a model for developing therapeutics intended for humans.

### AMD-Associated Changes of the Complement Transcriptome by Single-Cell RNA-seq in Human Retina and Choroid

**Figure 8:**
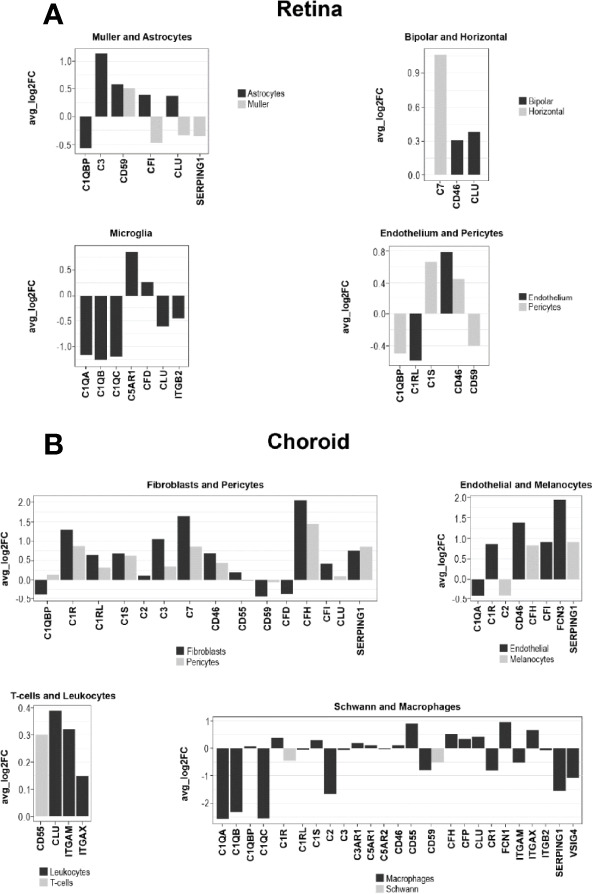
Log fold change (FC) of complement gene transcription within retina and choroidal cells from donors diagnosed with early AMD. **(A)** LogFC of Differentially expressed (DE) retinal genes between early AMD macula and normal macula were defined as a gene showing expression in at least 10% of cells within a cell type and a P ≤ 0.05. **(B)** LogFC of DE choroidal genes between early AMD macula and normal macula were defined as in A.

The species-related differences in complement necessitate a focus on human donor tissue to fully understand the role of complement in AMD pathology. To achieve this, we applied scRNA-seq to samples from early AMD cases and healthy donors. We clustered human retinal cells collected from human retina from donor eyes (**Table S1**) and performed analyses of complement gene expression, comparing early AMD macula vs normal macula and macula vs periphery in early AMD samples. For the comparison of early AMD vs. normal macula retina we identified several complement genes with significant differential expression for the retina (p≤0.05); most fold changes are small in magnitude (**Figure 8A** and **Data File S1**). The largest fold changes (FC) were found for *C1Q* which is down-regulated in microglia, *C3* which is up-regulated in astrocytes, and *C7* which is upregulated in horizontal cells. In microglia the anaphylatoxin receptor *C5AR1* and *CFD* was up-regulated and *ITGB2* was down-regulated. Membrane-bound complement inactivator *CD46* was upregulated in endothelium, pericytes and in bipolar cells. *CFI* was up-regulated in astrocytes and potential gliotic Müller cells, but down-regulated in homeostatic Müller cells. The inhibitor of MAC formation *CD59* was up-regulated in astrocytes and Müller cells and down-regulated in pericytes. The complement regulator *CLU* was up-regulated in astrocytes and bipolar cells and down-regulated in microglia and in Müller cells. Complement regulator *C1QBP* was down-regulated in both astrocytes and pericytes. In pericytes, *C1S*, which is required for *C1* activation, was up-regulated 1.6-fold.

To find regional expression differences for retina complement in AMD, we also compared early AMD macula vs. early AMD periphery (**Data File S1**). The largest differential expression between macula and periphery was found in astrocytes, where *C3* and *CFI* show about 2-fold lower expression in macula compared to periphery. *C3* showed higher expression in microglia in the macula. Other complement genes show fold change smaller than 1.5-fold.

Cells from the choroid were collected from the same donor eyes for single-cell complement analyses similar to retinal cells. In contrast to our results for retina, the choroid showed larger changes to 5-fold (**Figure 8B** and **Data File S1**). All the cell types in the choroid manifested transcriptional differences in one or more complement genes when comparing early AMD and normal macula sub-macular choroid (**Figure 8B** and **Data File S1**). Melanocytes had increased *SERPING1* and *CFH* expression. Macrophages showed a decline in *C1Q* (5-fold) and *SERPING1* (3.3-fold) and *CD59.* Increases in C*FH* expression were seen in early AMD sub-macular regions. In AMD donors, choroidal endothelial cells from the sub-macular region showed an increase in *CD46* and *FCN3* (3.8-fold). Pericytes showed increased expression of *C1R*, *C1S*, *CD46*, C*FH*, *C7*, *C3* and *SERPING1*. Choroidal fibroblasts from the sub-macular region had higher *C3*, C*FH*, *SERPING1* and C*FI* expression in early AMD. and a decrease of C*FD* (1.2-fold) in early AMD. Fibroblasts also expressed *C7* at higher levels than pericytes. Leukocytes, T and B cells had minimal differentially expressed (DE) complement transcripts.

Choroidal sub-macula and peripheral cells from early AMD donors were compared for potential clues to explain why the macula is the preferred site of AMD pathology. The largest differences between sub-macula and periphery (**Data File S1**) of the choroid in early AMD were present in Schwann cells, melanocytes, macrophages, endothelial, and fibroblasts. Schwann cells had less *CLU* and C*FD* expression in the sub-macular region. Macrophages from the sub-macula showed decreased *C1QB*, *CR1*, *C3* and increased C*FD*, *CD55*, and *FCN1*. Choroidal endothelial cells from the sub-macular region had a 2.8-fold increase of *FCN3*. Choroidal fibroblasts from the sub-macular region showed decreases in *CLU*, *SERPING1*, *C1R*, *C1S*, *C3*, *CD55* and increased C*FD* expression.

### Early and Late AMD-Associated Changes of the Complement Transcriptome by Bulk RNA-seq in Human Retina and RPE/Choroid

**Figure 9:**
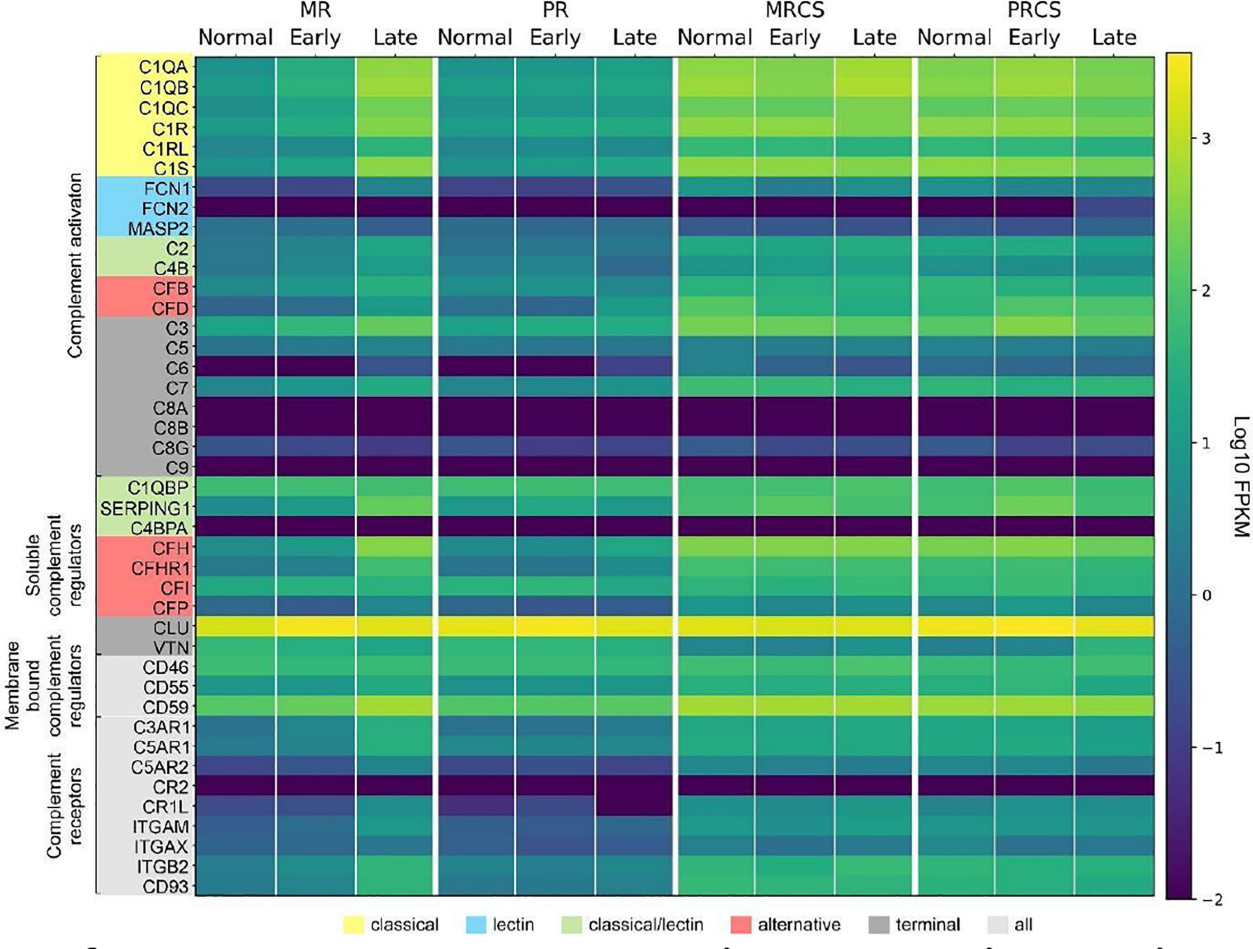
Heat map of complement gene expression determined using bulk RNA-seq for macular retina (MR), peripheral retina (PR), macular RPE/choroid/sclera (MRCS) and peripheral RPE/choroid/sclera (PRCS) from normal, early AMD and advanced AMD donors. Expression is measured in units of FPKM and color-coded on a logarithmic scale.

We next determined the difference in complement expression using bulk RNA-seq data obtained from retina and RPE/choroid/sclera tissue samples from normal and patients diagnosed with AMD. Because this dataset includes results from early- and late-stage AMD cases, it also allowed us to answer the question of whether trends in complement changes in early AMD are confirmed or even amplified with disease progression. In macular retina (MR), the majority of complement genes are up-regulated in late AMD (**Data File S2** and **Figure 9**). The largest FC are for all components of the C1 complex (*C1QA, C1QB, C1QC, C1R, C1S*) that recognizes antibody-antigen interactions and initiates the CP of complement activation. FC (late AMD versus normal) for these genes range from 67-fold for *C1Q* to 32-fold for *C1R*. Activators of the AP, *CFB* (8-fold) and *CFD* (16-fold) were also strongly upregulated. *FCN1*, an activator of the LP, was 17-fold upregulated in the MR of late AMD. Interestingly, the gamma subunit of C8 is expressed in our samples at very low levels and with little variation with respect to location or disease state while C9 is completely absent in our data (**Figure 9**). The next-highest fold changes in MR are the soluble negative regulators of complement activation, *CFH* up-regulated 63-fold and *SERPING1* up-regulated 37-fold in late AMD. This trend was slightly visible already in early AMD retinae and is in line with *CFH* upregulation in pericytes determined by scRNA-seq as well. Of the membrane-bound inhibitors of complement activation, only *CD59* is significantly up-regulated (5-fold in late AMD). *CLU*, which inhibits formation of the terminal C5b-9 complex, is expressed at a high level irrespective of disease stage or location (**Figure 9**). *VTN*, another antagonist of C5b-9 complex formation, is expressed at low levels and is the only inhibitor down-regulated (3-fold) in MR of late AMD patients. Also, *CFP* is up-regulated 4-fold suggesting a more active amplification loop of C3 convertase formation in AMD. Other genes with significant up-regulation in MR in late AMD include the anaphylatoxin receptors, *C3AR1*, *C5AR1* and *C5AR2* (15-24-fold), the integrins *ITGAM*, *ITGAX*, *ITGB2* (3-20-fold), and the adhesion molecule *CD93* (20-fold). In peripheral retina (PR), some up-regulation is observed in late AMD, but with much smaller fold-changes (**Data File S2** and **Figure 9**).

In macular RPE/choroid/sclera (MRCS) and peripheral RPE/choroid/sclera (PRCS) bulk data, patterns of expression for most complement genes do not depend strongly on disease state. For some of the deviations the interpretation is not clear. For example, *CFD* shows modest down-regulation with AMD in MRCS and up-regulation in PRCS. Because these samples contain RPE cells and, most importantly, a large proportion of scleral cells in addition to choroidal cells, no cross-comparison was performed with the data from our scRNA-seq approach to avoid over- or misinterpretation of the data.

These observations thus partially confirm our findings from the scRNA-seq approach and, importantly, suggest a robust perturbation of complement homeostasis in the MR in advanced AMD which is not present in the retina periphery, choroid MRCS and PRCS.

## Discussion

Understanding how the local complement homeostasis in the retina and supporting tissues is changing in the course of diseases like AMD is key to identify appropriate targets and optimal timing for a successful therapeutic intervention. Anderson et al. published a comprehensive analysis of complement expression in human RPE, choroid and retina (17). Their analysis was done on tissue layer lysates as opposed to the current study which focused on single cells from these layers. They characterized the choroid as the predominant source of soluble regulators, SERPING1 and C4BP. The membrane regulators, CD55 and CD46, were also higher expressed in the choroid than in RPE or retina. C3 and the terminal pathway genes, C5 and C7, expression was localized within the choroid as well. While the regulators CLU and VTN were expressed in all three layers. LP expression was very low in all three layers. Tissue lysates from several donors with AMD were also assayed by quantitative PCR but no significant differences were detected between normal and AMD eyes (17). Their overall results concluded that the choroid was the predominant source of CP and AP components, rather than the RPE or retina. Our study extends the Anderson et al. study to single cell expression of the complement genes in normal retina, RPE and choroid as well as early AMD retina and choroid (17).

### A Refined Map of Complement Expression in Retina, RPE and Choroid

C1q is the recognition protein for the CP and it must complex with both C1r and C1s to activate the CP (1). The retina showed distributed transcription of *C1Q*, *C1R* and *C1S* with *C1Q* expressed in microglia and *C1R*/ *C1S* expressed in both astrocytes and pericytes (**Figure 1A**). Of note, *C1Q* was expressed by almost all sequenced microglia. Astrocytes and pericytes expressed also most of the soluble complement regulator *SERPING1*, a dissociator of the C1 complex (**Figure 1A**). Thus astrocytes/pericytes appear to have opposing roles as activators and inhibitors of the CP. Interestingly, *SERPING1* is significantly downregulated in astrocytes in macula vs periphery in early AMD (**Data File S1**).

In the normal choroid, *C1Q* is solely expressed in macrophages which is in line with findings for retinal microglia (**Figures 1A, C**). Choroidal pericytes and fibroblasts express both *C1R* and *C1S* (**Figure 1C**). Multiple cell types in the choroid including fibroblasts, pericytes, Schwann cells and melanocytes show robust *SERPING1* expression. According to scRNA-seq results, the RPE has minimal transcription of CP activators (**Figure 1B**).

The initiators of the LP pathway, FCN1-3 and MASP1, showed no expression in the normal retina, choroid and RPE suggesting the LP pathway is inactive in all healthy layers.

The C3 convertase is short-lived with a half-life of about 90 seconds requiring stabilization to assure efficient host defense (1). Properdin (gene ID: CFP) stabilizes the alternative C3 convertase and is solely expressed in macrophages of the choroid but is not expressed in RPE and retina (**Figure 1**). While an antagonistic inactivator of C3, FI, was primarily contributed by Müller glia, an important macroglial cell type in the retina (**Figure 1A**).

The late-acting complement components of the terminal pathway, including C7, assemble into the terminal complement complex to form either a cell membrane pore that induces cell lysis or a soluble sC5b-9 complex with multiple functions. Except for C7, the other components of the terminal pathway were not detected in healthy cells. *C7* transcripts were expressed in both normal retina and choroid, albeit confined to the horizontal cells in the retina and fibroblasts in the choroid. *C7* transcripts translate into a 91 kDa protein that we demonstrated to be present in the healthy neuroretina and RPE by western blotting (**Figure 7B**). C7 has a prominent cathepsin D cleavage sites leading to lysosomal protein degradation fragments, which were additionally detected in all retinal cell types (61). The functional relevance of C7 cleavage products however is still unknown. Immunostaining showed a uniform distribution of C7 in the macular and peripheral human retina. This study, thus, provides new insights into the retinal localization of C7 as the only late complement protein expressed in the neuroretina (**Figure 2**). Additionally, *C7* is upregulated in both astrocytes and horizontal cells in early AMD.

Terminal complement components, namely the C5b-9 complex, have previously been detected only in the Bruch's membrane/choroid complex in aged healthy controls (17, 62). Pore formation is triggered *via* the conversion of C5 to C5b by surface-bound C5 convertases and subsequent local formation of C5b6. The next step, binding of C5b6 with C7 must occur rapidly to prevent release of C5b6 from the membrane surface. If C7 concentrations near the site of complement activation are limiting, the stable bimolecular C5b6 complex dissociates from the C5 activating complex and accumulates in solution (63). If this C5b6 complex subsequently encounters C7, fluid‐phase C5b‐7 is formed, and this complex can lyse normal cells at a different location from the initial site by ‘reactive lysis’ (64). Therefore, it is advantageous to have local retinal horizontal and choroidal fibroblasts expressing C7 so that its presence can prevent diffusion of C5b6 from the cell membrane.

The soluble inhibitors for the C5b-9 terminal complex, CLU and VTN, had different cellular expression patterns. In the retina, all cells expressed *CLU* but in the choroid its expression was limited to mast, endothelial, melanocytes, Schwann, pericytes and fibroblasts. RPE also had robust *CLU* expression activity. *VTN* was expressed in rods, cones and horizontal cells. Very low *VTN* expression was detected in both choroid and RPE. The robust expression of *VTN* and *CLU* in healthy cells is important to decrease the deposition of C5b-9 onto their surface. Importantly, *CLU* was upregulated in both ganglion and horizontal cells in the macula of early AMD.

### Implications of Cell Type-Specific Protein Signatures for Selected Complement Components

To infer functional implications of a particular complement component in retinal immune homeostasis, one must ask where the respective protein accumulates in the tissue, especially if, as it is the case with most complement components, the protein of interest is secreted. Our protein expression data of selected complement components in the healthy adult human retina showed for the first time (i) an intraretinal localization of C1s and C7, (ii) spatial differences for C3 detection in the macula and periphery, and (iii) a FI colocalization with microglial cells, indicating a physiological function of the complement system in the human retina.

C1s, is one of the first proteolytically active components of the CP. The assembly of the classical C3 convertase (C4bC2a) requires the activity of C1s. We detected the heavy chain of C1s mainly in Müller and vascular cells purified from healthy retinal tissue (**Figure 4B**). Human retinal immunostaining showed associated C1s deposition in the macular and peripheral areas in the inner plexiform, ganglion cell, and compartments of the photoreceptor layers (**Figures 4C, D**). The punctate C1s-positive structures did not overlap with the tested Müller cell, amacrine or ganglion cell markers or nuclei, but seemed to be allocated to cell surfaces or the intercellular space. According to the human cell atlas, C1s is mainly localized in the nucleoplasm and additionally in the cytoplasm (65). In line with present findings, we recently detected C1s heavy chain in the photoreceptor and ganglion cell layer of healthy, photodamaged and post-ischemic murine retinas (48, 66).

C3 is the central complement protein and all complement pathways converge at the level of C3 activation. It is expressed differently in the RPE, retina and choroid. *C3* mRNA was modestly expressed in retinal microglia and astrocytes, robustly expressed in choroidal fibroblasts and minimally expressed in RPE cells (**Figure 1**). We detected C3 protein deposition in healthy retinas (**Figures 6C–F**). In the retinal periphery, C3 protein colocalized with Müller cells, endothelium and with cells in the ganglion cell layer (**Figure 5D**). In the macular region, C3 protein was detected in the outer plexiform layer and on cone photoreceptors (**Figure 5C**). Previous work also reported C3 immunoreactivity for a subset of cone photoreceptors approximately 1.5 mm peripheral to geographic atrophy lesions but not in healthy retinas (67). The outer plexiform layer and photoreceptor-associated C3 deposition in healthy tissue was largely restricted to the macular region in our study (**Figure 5C**). Comparison of our results with other studies is difficult as in most cases it is not clearly specified which retinal regions were used for staining in healthy controls and the ganglion cell layer was never imaged (17, 67, 68). Our C3 protein results for the healthy retina add to the current knowledge on C3 immunoreactivity in Bruch's membrane/choroid.

While the liver is the predominant source of circulating C3 (69), it has been shown to be synthesized by immune and nonimmune cells such as lymphocytes, neutrophils, epithelial, and endothelial cells (9, 70, 71). In some cases, the accumulation of intracellular C3 can aggravate tissue damage, while in others it can be protective against cytokine induced death (72–75). There is good evidence that intracellular complement provides tissue specific protection against distinct stimuli such as injury and in some cases functions in cell metabolism (76–78). Kulkarni et al. published elegant work on C3 biosynthesis in human airway epithelial cells that is augmented during times of stress and acts as a cytoprotectant (71). The same study found increased intracellular C3 in airway epithelial cells in end-stage lung disease due to cystic fibrosis or chronic obstructive pulmonary disease. In our study choroidal fibroblasts demonstrated an increase of C3 expression in early AMD, potentially for a protective effect. It is unknown how intracellular C3 stores in the choroid and retina are modulated and whether altering these stores is deleterious or protective.

Complement responses are tightly regulated. The damaging C3 cleavage product, C3b, is inhibited by FH and FI in the fluid phase and on membrane surfaces by the receptors CD55 and CD46. Retinal endothelial cells express *CFH* and *CFI*, while Müller glia express *CFI*. Also, the choroid had robust expression of *CFH* and *CFI* from endothelial cells and fibroblasts. Western blots associated FI mainly with the retinal microglial cell population (**Figure 6F**) and to a lesser extent to RPE/choroid. This corresponded with FI immunostaining in the plexiform layers in the adult healthy retina, and with overlapping microglial staining in the inner plexiform layer (**Figures 6G, H**) (79). In addition, partial overlap of FI staining with the Müller cell marker glutamine synthetase was observed, which is consistent with their single-cell mRNA profile (**Figures 2**, **6G**). In line with these findings, previous publications localized FI immunoreactivity mainly to the inner retina (17).

FH is the major complement inhibitor of the AP. Consistent with previous reports (17, 80), we detected FH protein in the RPE/choroid and at vessels in the healthy human retina. We could not identify spatial differences between macular and peripheral retinal tissue. This suggests a role in maintaining retinal immune privilege at the inner and outer retinal blood barrier rather than intraretinal activity.

Almost half the retinal cell types expressed *CD55* or *CD46* which inactivates C3b and C4b in the presence of FI. Not detectable expression of *CD55* and *CD46* in ganglion cells, astrocytes, microglia, rods, cones and Müller glia makes these cell types more susceptible to damage from C3b and C4b binding. In addition, every choroidal cell type expressed either *CD46* or *CD55*, providing broad protection from C3b and C4b. RPE expressed *CFH* and *CFI* and modestly expressed the receptors *CD46* and *CD55* that provide a reasonable barrier against complement damage. Our finding of less *CD46* and *CD55* expression on normal rods and cones might be the reason C3 is able to accumulate on photoreceptor surfaces in AMD.

### ScRNA-seq Refines Our Understanding on the Cellular Contribution to Complement Changes in AMD

While only very moderate changes in complement expression were observed in early AMD for retinal cell types, our choroidal scRNA-seq findings demonstrated increased expression of the secreted complement components *CFI, C1R, FCN3, CFH, C3, C7* and *SERPING1. C3* was increased in fibroblasts (2-fold) and pericytes (1.3-fold) and *C7* was increased 3-fold in fibroblasts and 1.8-fold in pericytes. *FCN3* had minimal expression in normal endothelial cells but increased 3.8-fold in early AMD. Ficolins serve as recognition molecules for the LP and activate the MBL-associated serine protease family, MASPs. Importantly, *FCN3* is a primate specific gene and only exists as a pseudogene in mice (81). It has been reported to bind to apoptotic Jurkat cells promoting C3 and C4 activation (82). The membrane regulator CD46 was ubiquitously expressed in normal choroid and showed higher expression in most of the same cell types in early AMD (fold change ranging from 1.1-2.6). The rise in *CD46* expression could be a compensatory response to the enhanced secreted complement component expression. In early AMD, *CFH* also showed large increases in early AMD in macrophages, pericytes, and fibroblasts - most likely to offset increased C3b.

Our comparison of the sub-macula vs peripheral choroid in early AMD identified significant increases in *FCN3* expression in endothelial cells of the sub-macula (3-fold) than in peripheral choroid. Interestingly, FCN3 was reported to be elevated in the vitreous of eyes with proliferative diabetic retinopathy along with increased VEGF suggesting a collaboration between FCN3 and VEGF to stimulate inflammation and angiogenesis (83). Other differences in choroidal macular complement expression from early AMD eyes included a 3-fold decrease of *CFD* and 1.5-1.6-fold decrease of *CLU* in Schwann cells and fibroblasts as well as a 1.4-fold *FCN1* and 1.3-fold C*FD* increase in macrophages compared to their cellular counterparts from peripheral AMD choroid.

Currently, there are two competing theories of the tissue layer responsible for the initiation of AMD. One theory implicates RPE cell dysregulation as the starting location because drusen and RPE pigmentary changes often precede advanced AMD stages (84). Alternatively, more recent evidence cites the choriocapillaris as the initiating site due to capillary dropout, a hallmark of early AMD (85). In line with this, Lutty et al. reported attenuation of the vascular supply in the submacular region with overlying normal RPE in early AMD (86). The vascular diminution was confined to the macula and absent in the periphery providing strong evidence that the choroid, not the RPE, could be the initiation site of AMD (81). Our complement results in the macula of early AMD provide further evidence (i) that there are choroidal expression differences confined to the macula region and (ii) that the retinal cell complement expression signature is not as dramatically changed as observed in the choroid which implies that (iii) the disruption of the local complement homeostasis in early AMD is driven by the choroid rather than retinal cell types. Since our analysis did not include RPE, we cannot determine its contribution to the pathology.

### Bulk RNAseq Confirms Early Changes of Complement Expression That Are Accelerated With AMD Progression

While bulk RNA-seq data are relatively insensitive to gene expression changes limited to specific cell types (which might provide important clues to mechanisms of disease initiation), this approach does provide a global picture of large-scale changes in expression at the tissue level. In particular our data for macular and peripheral retina reveal that AMD progression is associated with increasing up-regulation of a number of complement genes associated with the CP and AP and therewith validate findings from our scRNA-seq approach, and that these changes are largely restricted to the macular retina (**Figure 8** and **Data File S2**).

In moving from normal through early and into late-stage AMD there is progressive up-regulation in the macula retina of complement genes associated with the CP, including all components *C1Q*, the *C1Q*-associated activators *C1R* and *C1S*, *C2* and *C3*, and the initial members of the membrane attack complex *C5*, *C6* and *C7*. The soluble complement inhibitors *CFH* and *SERPING1* are also up-regulated, as well as the membrane-bound regulator of the C5b-9, *CD59*, perhaps reflecting regulatory feedback accompanying complement activation. The up-regulation of anaphylatoxin receptors (*C3AR1*, *C5AR1*, *C5AR2*) is most pronounced in late AMD, as is the expression of *ITGAM* and *ITGB2*, implying the activation of phagocytosis related to complement-coated particles in late AMD. This pattern of progressive up-regulation of complement genes is only weakly observed in the peripheral retina.

In contrast, the expression levels of complement genes in the macular and peripheral RPE/choroid/sclera (RCS) are similar to the highest levels observed in the macular retina, and are relatively uniform with respect to disease stages, with a small number of differentially-expressed complement genes (**Data File S2**). Interestingly all the DE genes in macula RCS show down-regulation in late AMD, while genes in the periphery RCS are mainly up-regulated. In particular, expression of *CFD* is down-regulated in late AMD in MRCS, and up-regulated in PRCS. We note that the expression of the terminal C5b-9 components *C8* and *C9* are largely absent in all of our RNA-seq data, but are observed at the protein level which may reflect proteins transported from the choroid blood supply.

A recent study of nine complement genes in normal eyes and eyes with early, intermediate and advanced AMD partially recapitulates our results (87). Their results for macula retina showed a complex pattern of regulation for most of the nine complement genes examined when moving from early to intermediate AMD and partially agree with our results. However, their results are almost in complete accord with our findings for late-stage AMD, showing up-regulation of *C1QA*, *C3*, *C4B*, *CFB*, *CFD*, *CFH* and *MASP1* in late AMD with respect to control. In contrast, we did not reproduce their finding of significant up-regulation of *CFI* and down-regulation of *CFP*. Our results for RCS similarly showed higher average expression for most complement genes compared to macula retina. Additionally, both studies showed down-regulation of *CFD* in the RPE/choroid. Both studies found no expression for terminal MAC component *C9*.

Our bulk data results suggest that even though the initiation of AMD may not depend strongly on changes in retinal cells, the macula retina is still the principal site of complement activation as the disease progresses.

### Species Differences in Retinal Complement Expression Complicate the use of Animal Models in AMD Research

A thorough comparison of the cellular landscape of complement expression in the retina across species is important to assess the usability of animal and specifically mouse models to investigate pathological mechanisms of AMD for the development of human therapies. For that reason, we compared results from the present study on human retina with previous results collected from its mouse counterpart and, indeed, found considerable differences (48). These low levels in mouse retina (but significant detection in the human retina) should be considered when investigating the mouse AP. Finally, there is the striking difference regarding membrane bound complement regulator expression. Absence of CD46 and CD55 in mouse retina would suggest a different pathway for inactivating both C3b and C4b at the cell membrane surface. Of course, these expression differences exist under a normal physiological state and could change in the setting of disease.

Our results provide strong evidence of complement synthesis in the healthy retina, RPE and choroid. The body of evidence suggests that local complement expression impacts all cell types and that complement’s functions in the tissue environment could go beyond its role in the innate system (88). Accordingly, there is an urgent need to further investigate alternative complement functions in the human eye. Multiple publications provide evidence for complement orchestrating normal cell and organ development even in the immune privileged central nervous system (89, 90). These include direct tissue repair and regulation of basic processes of the cell, particularly in metabolism (77). A receptor for C1q has been identified on mitochondria which in the presence of intracellular C1q mediates mitochondrial ROS production (91). A direct link between C3a and downregulation of proteasome activity was reported in human RPE cells from older individuals, suggesting a link with intracellular protein longevity (92). Our data identified multiple complement genes expressed under normal physiological conditions that must have biological functions that maintain integrity of the retina, RPE and choroid. Further studies are necessary to investigate complement function for its role in homeostasis to provide a foundation for AMD clinical trials evaluating treatment with complement inhibitors [reviewed in (36–38)].

Limitations of our study include a focus on European American donors and a limited number of disease donors for single cell analysis.

## Materials and Methods

### Human Donor Eyes

Tissues were obtained from human eye donors at different institutions, as indicated below. The Institutional Review Boards of each institution approved the respective use of human tissues.

### Study Subjects and scRNA-seq

Samples were taken from macular and peripheral regions of the retina from two normal donor eyes (**Table S1**, samples 18-1077-W, 18-1132-P) at UAB and used in scRNA-seq studies at UPenn as described in (93). Briefly, raw counts were converted to log-normalized expression values with a scale factor of 10,000 UMIs per cell, the 2,000 most variable genes across all cells were identified, and the cells were clustered using the DESC algorithm (94). Eleven major retinal cell types were identified using this data, providing cell-type-specific expression profiles for complement genes.

Additional samples were obtained from three donor eyes (**Table S1**, samples 20-1166-W, 20-1438-W, 20-1484-W) at UAB and used in scRNA studies at UPenn. All donor eyes were collected within 6 hours postmortem and characterized for presence of AMD and other pathology by author C.A.C. Following removal of the anterior chamber and vitreous, the eyecup was immersed in oxygenated Ames media. Relief cuts were made in the posterior eyecup to expose the tissue and 8mm punches were obtained from macular retina and temporal (peripheral) retina and carefully isolated from the retinal pigment epithelium. The choroidal layer was isolated similarly. The isolated tissue layers were dissociated with activated papain (Worthington Biochemical Corp.) as previously optimized to obtain a high percentage of viable cells. After dissociation, magnetic bead-based removal of dead cells (Miltenyi Biotec) was used to reach the optimum target for viability of 85-95% per sample. Viability was determined by FACS sort or by staining an aliquot of the dissociated cells with trypan blue, 0.4% (Sigma-Aldrich). Single cell transcriptome libraries were prepared by using 10xGenomics Single Cell 3’ biased v2 kit according to the company’s manual. The constructed single cell libraries were sequenced by HiSeq 2000 sequencer (Illumina, Inc., San Diego, CA, USA) with total reads per cell targeted for a minimum of 50,000. Raw base call (BCL) files were aligned to human genome reference GRCh38-2020-A and processed with 10x Genomics Cell Ranger 3.1.0 to produce gene count matrices for each sample. For each sample replicate, we performed initial quality control using Cell Ranger (Version 3.1.0). Then, we further filtered the data using Seurat (version 3.0). A cell was retained in downstream analyses if it meets the following criteria (1): More than 200 genes are detected (2); Total number of UMIs is between 1,000 and 25,000. Retina and choroid cells were separated into two datasets and analyzed separately from each other. Each dataset was processed using Seurat 3.0, a statistical framework to combine cell gene expression profiles measured by scRNA-seq (95). Expression data was normalized by dividing the counts for each feature of a cell by the total counts for that cell and multiplying the result by a scale factor of 10,000; this was then transformed to a natural-log scale. Gene expression levels were normalized using the 2,000 most variable features in the datasets as identified by Seurat’s *FindVariableFeatures* function. Cell clustering was completed using Seurat’s shared nearest neighbor (SNN) modularity optimization-based clustering algorithm. This clustering was performed and evaluated using a range of resolution values, with a resolution of 1.5 being selected for the choroid cells and 0.2 for the retina cells. Differentially expressed genes for the generated clusters were compared to known choroid and retina cell type markers to identify and label cell-types, and cell-type-specific expression profiles for complement genes were determined. Eleven major retinal and ten major choroidal cell types were identified in this analysis. Since no RPE cells were recovered in this study, sc-RNA-seq data from Voigt et al. was reanalyzed and used to supplement our data with complement gene expression levels for that single cell type (51).

### Bulk Tissue Processing and Data Generation

#### Eye Collection and AMD Assessment

This study utilized 14 pairs of eyes from non-diabetic Caucasian donors 69-95 yr of age (84.73 yr ± 5.53 yr; 7 males and 7 females) at a death-to-preservation interval of < 6 hr. Ocular health histories were not available. Eyes were opened by eye bank recovery personnel using an 18 mm diameter corneal trephine, followed by a snip to the iris to facilitate penetration of preservatives into the fundus. The left eye was preserved in RNAlater (Qiagen) at 4°C. Left eyes were shipped on wet ice *via* overnight courier to University of Pennsylvania where they were processed for bulk RNA sequencing upon arrival.The right eye was preserved in 2% glutaraldehyde and 1% paraformaldehyde in 0.1M phosphate buffer at 4°C. It was assessed for maculopathy at UAB by internal inspection using a dissecting scope (Nikon SMZ-U) with oblique trans- and epi-illumination in consultation with an MD medical retina specialist, ex vivo multimodal imaging of excised 8 mm diameter macular punches using digital color photography and spectral domain optical coherence tomography volume scans (SD-OCT; Spectralis, Heidelberg Engineering) with a custom tissue holder (co-author JDM), and high-resolution epoxy-resin histology, as described. The definition of AMD used in this study was the presence of one large druse (>125 µm in diameter) in the macula or severe RPE changes in the setting of at least one druse or continuous basal linear deposit, with or without the presence of neovascularization and its sequelae. Eyes with geographic atrophy had at least one region 250 µm in diameter lacking a continuous RPE layer (but possibly containing ‘dissociated’ RPE). Unremarkable eyes were those lacking characteristics of AMD or other chorioretinal disease as discernible in either histology or ex vivo imaging; these served as comparison eyes.

#### Sequencing and Analysis of Bulk Data

RNA for the eye tissues was extracted using the AllPrep DNA/RNA Mini Kit (Qiagen). Extracted RNA samples underwent quality control assessment using R6K ScreenTape on a 2200 Tapestation (Agilent, Santa Clara, CA, USA) and were quantified using Qubit 2.0 Fluorometer from Life Technologies (Grand Island, NY). All RNA samples selected for sequencing had an RNA integrity number of ≥8. The Strand-specific RNA library was prepared from 100 ng total RNA using the Encore Complete RNA-seq library kit (Nugen Technologies, Inc., San Carlos, CA, USA) according to the manufacturer’s protocol. RNA-sequencing was performed at the Center for Applied Genomics at the Children’s Hospital of Philadelphia per standard protocols. The prepared libraries were clustered and then sequenced using HiSeq 2000 sequencer (Illumina, Inc., San Diego, CA, USA) with four RNA-seq libraries per lane (2 × 101-bp paired-end reads). The RNA-seq data were aligned to the hg38 reference genome using GSNAP (version 2016-06-30) with known splice sites (SNP file build 147) taken into account. In order to eliminate mapping errors and reduce potential mapping ambiguity owing to homologous sequences, several filtering steps were applied. Specifically, we required the mapping quality score of ≥30 for each read, reads from the same pair were mapped to the same chromosome with expected orientations and the mapping distance between members of the read pair was 200,000 bp. Quality control analysis of the aligned data was performed using program RNA-SeQC. All subsequent analyses were based on filtered alignment files. Per-gene counts were generated from the GSNAP alignments using the HTSeq-count program (version 0.6.0) using default ‘union’ mode and the HG38 reference genome.

### *In Situ* Hybridization

#### Human Retina Tissue Preparation

Within 6 hours of death, posterior poles were fixed in freshly made 4% paraformaldehyde/0.1M phosphate-buffered (PB, pH 7.4, Thomas Scientific, LLC, Swedesboro, NJ, USA) overnight, then washed and stored in 1% paraformaldehyde/0.1 M PB at 4°C. Eyes were shipped on wet ice *via* overnight courier to University of Pennsylvania. This study conformed to Institutional Review Board regulations for use of human tissues at University of Alabama at Birmingham (UAB) and at University of Pennsylvania. Dissection of retina/choroid/sclera was performed as described (96). Retina tissues were cryoprotected in increasing concentration of sucrose as described (97) followed by embedding in OCT (Tissue Tek, Sakura Finetek USA, Torrance, CA, USA) and immediately snap-frozen in ice-cold 2-methylbutane. The frozen tissue was sliced at 10 µm in the cryostat and stored at -80°C.

#### RNA-FISH Protocol

Single molecule RNA *in situ* hybridization (RNA-FISH) was carried out with the RNAscope® Multiplex Fluorescent Reagent Kit v2 (Advanced Cell Diagnostics, INC, Newark, CA, USA). Probes were used for this study are: Hs-AIF1-C3 (#433121-C3), Hs-ONECUT1 (#490081), Hs-CD34-C2 (#560821-C2), Hs-CFH (#428731), Hs-CFI (#421921), Hs-CLDN5-C2 (#517141-C2), Hs-RLBP1-C2 (#414221-C2), Hs-FOS (#319901), Hs-JUN (#470541), Hs-C3AR (#461101), Hs-ITGAX (#419151), Hs-ITGB2 (#480281), Hs-VSIG4 (#446361), Hs-C3 (#430701), Hs-C7-C3 (#534791-C3), Hs-CD46 (#430151), Hs-CD55 (#426551), Hs-CFD (#420831). Positive control RNAscope® 3-plex probe (#320861) and RNAscope® 3-plex negative control probe (#320871) were used (data not shown) for each experiment. In addition, probe diluent (#300041) was used for negative control.

### Proteomic Profiling of MACS Enriched Retinal Cell Types

#### Tissue Collection

Samples for proteome profiling were isolated from a set of five donor eyes. The Institutional Review Board at University of Regensburg approved the use of human tissues for this purpose. One eye per donor from five non-diabetic Caucasian donors 59-89 yrs of age (4 males and 1 female) at a death-to-experimentation interval of < 30 hr were included in this analysis (**Table S1**). Ocular health histories were not available. Eyes were opened by eye bank recovery personnel using an 18 mm diameter corneal trephine and stored on ice for transfer to the laboratory for further processing.

#### Cell Purification From Human Donor Retina

Retinal cell types were enriched as described previously using magnetic-activated cell sorting (MACS) (98). Briefly, retinal punches (6 mm in diameter, centered over the fovea and for comparison, the peripheral punch was performed at 1 mm from the macular punch and inferiorly in relation to it) were treated with papain (0.2 mg/ml; Roche Molecular Biochemicals) for 30 minutes at 37 °C in the dark in Ca^2+^- and Mg^2+^-free extracellular solution (140 mM NaCl, 3 mM KCl, 10 mM HEPES, 11 mM glucose, pH 7.4). After several washes and 4 minutes of incubation with DNase I (200 U/ml), retinae were triturated in extracellular solution (now with 1 mM MgCl_2_ and 2 mM CaCl_2_). To purify microglial and vascular cells, the retinal cell suspension was subsequently incubated with anti-mouse/human ITGAM (alias CD11B) and anti-human CD31 microbeads according to the manufacturer’s protocol (Miltenyi Biotec, Bergisch Gladbach, Germany). The respective binding cells were depleted from the retinal suspension using large cell (LS)-columns, prior to Müller cell enrichment. To purify Müller glia, the cell suspension was incubated in extracellular solution containing biotinylated anti-human CD29 (0.1 mg/ml, Miltenyi Biotec) for 15 minutes at 4°C. Cells were washed in an extracellular solution, spun down, resuspended in the presence of anti-biotin ultra-pure MicroBeads (1:5; Miltenyi Biotec,) and incubated for 10 minutes at 4°C. After washing, CD29+ Müller cells were separated using LS columns according to the manufacturer’s instructions (Miltenyi Biotec). Cells in the flow through of the last sorting step - depleted of microglia, vascular cells and Müller glia - were considered as the neuronal population. RPE was obtained by scraping from the sclera of the punched-out piece of tissue after the retina had been removed, so those samples inevitably contained cells of the underlying choroid. Therefore, those samples are referred to as RPE/choroid throughout the manuscript.

#### LC-MS/MS Mass Spectrometry Analysis

LC-MS/MS analysis was performed as described previously on a Q-Exactive HF mass spectrometer (Thermo Fisher Scientific Inc., Waltham, MA, U.S.A.) coupled to an Ultimate 3000 RSLC nano-HPLC (Dionex, Sunnyvale, CA) (99, 100). Briefly, 0.5 μg sample was automatically loaded onto a nano trap column (300 μm inner diameter × 5 mm, packed with Acclaim PepMap100 C18. 5 μm, 100 Å; LC Packings, Sunnyvale, CA) before separation by reversed phase chromatography (HSS-T3 M-class column, 25 cm, Waters) in an 80 minutes non-linear gradient from 3 to 40% acetonitrile (ACN) in 0.1% formic acid (FA) at a flow rate of 250 nl/min. Eluted peptides were analysed by the Q-Exactive HF mass spectrometer equipped with a nano-flex ionization source. Full scan MS spectra (from m/z 300 to 1500) and MS/MS fragment spectra were acquired in the Orbitrap with a resolution of 60,000 or 15000 respectively, with maximum injection times of 50 ms each. Up to ten most intense ions were selected for HCD fragmentation depending on signal intensity (TOP10 method). Target peptides already selected for MS/MS were dynamically excluded for 30 seconds. Spectra were analyzed using the Progenesis QI software for proteomics (Version 3.0, Nonlinear Dynamics, Waters, Newcastle upon Tyne, U.K.) for label-free quantification, as previously described (98). All features were exported as a Mascot generic file (mgf) and used for peptide identification with Mascot (version 2.4) in the UniProtKB/Swiss-Prot taxonomy mouse database (Release 2017.02, 16871 sequences). Search parameters used were: 10 ppm peptide mass tolerance, 20 mmu fragment mass tolerance, one missed cleavage allowed, carbamidomethylation set as fixed modification, and methionine oxidation, asparagine or glutamine deamidation were allowed as variable modifications. A Mascot-integrated decoy database search calculated an average false discovery rate (FDR) of < 1%.

#### Western Blot

Cell pellets of enriched cell populations from retinal punches were dissolved in reducing Laemmli sample buffer, denatured and sonicated. Neuronal protein extraction reagent (Thermo Fisher Scientific, Braunschweig, Germany) was added to the neuron populations. Samples were separated on a 12% SDS-PAGE. The immunoblot was performed as previously described (101). Detection was performed with primary and secondary antibodies diluted in blocking solution (**Table S2**). Blots were developed with WesternSure PREMIUM Chemiluminescent Substrate (LI-COR, Bad Homburg, Germany). To validate specificity of the antibodies, all of them were tested on human serum and purified proteins as positive control and human serum depleted for the respective complement factor as negative control (**Table S3**).

#### Immunofluorescence Labeling

To stain for complement components in the macular and peripheral retina, human eyes (postmortem time < 8 hours) were cryosectioned. The research complies with the human research act (HRA) stating that small quantities of bodily substances removed in the course of transplantation may be anonymized for research purposes without consent (HRA chapter 5, paragraph 38, Switzerland). Before sectioning, the eyes were immersion-fixated with 4% paraformaldehyde (PFA) for 48 hours. Thereafter, the central part of the eye cup containing the optic nerve head and the macula including the underlying RPE, choroid and sclera was dissected. The tissue was submitted to cryoprotection, embedded in OCT and cut into 20 µm thick sections. Retinal detachment from the RPE is an artifact commonly observed in cryosections

Retinal sections were permeabilized (0.3% Triton X-100 plus 1.0% DMSO in PBS) and blocked (5% normal donkey serum with 0.3% Triton X-100 and 1.0% DMSO in PBS) for 2 h at room temperature. Primary antibodies were incubated overnight at 4°C. Sections were washed (1% bovine serum albumin in PBS) and incubated with secondary antibodies (2 h at room temperature). Cell nuclei were labeled with DAPI (1:1000; Life Technologies). Control experiments without primary antibodies showed no nonspecific labeling. Images were taken with a custom-made VisiScope CSU-X1 confocal system (Visitron Systems, Puchheim, Germany) equipped with high-resolution sCMOS camera (PCO AG, Kehlheim, Germany).

#### Quantification and Statistical Analysis

Statistical analyses were performed using Prism (Graphpad Software, San Diego, CA, USA). In most of the experiments in the present study results from 4 to 5 biological replicates were collected. Since this low number of input values does not allow an appropriate estimation about a normal Gaussian distribution, significance levels were determined by the non-parametric Mann-Whitney U test unless stated otherwise. All data are expressed as mean ± standard error (SEM) unless stated otherwise. Detailed information about specific n-values, implemented statistical tests and coding of significance levels are provided in the respective Figure legends.

## Data Availability Statement

The datasets presented in this study can be found in online repositories. The names of the repository/repositories and accession number(s) can be found below: GEO, accession IDs: GSE188280, GSE155154, GSE155288.

## Author Contributions

Conceptualization: DP, AG, ML, DS. Methodology: RZ, JB, DS, YJ, MK, JH, LK, AP, NS, VE, US-S, TS, SH, PG, MM, JM, CS. Investigation: RZ, JB, YJ, DS, MK, LK, AP. Visualization: RZ, JB, YJ, MK, LK, AP. Supervision: DP, AG, ML, DS. Writing—original draft: RZ, JB, YJ, DP, AG, ML, DS. Writing—review & editing: RZ, JB, YJ, LK, SH, CC, DP, AG, ML, DS. All authors contributed to the article and approved the submitted version.

## Funding

This project was supported by Deutsche Forschungsgemeinschaft DFG‐GR 4403/5-1 (AG), ProRetina Foundation Germany Pro-Re/Seed/Kaplan-Grosche.8-2019 (AG, LK), Deutsche Forschungsgemeinschaft DFG-PA 1844/3-1 (DP), Deutsche Forschungsgemeinschaft DFG-HA 6014/5-1 (SH), Support Sight Foundation (TSSF) (DS), and National Institutes of Health grant R21EY031877 (ML), P30 EY003039 (UAB), R01EY030192 (ML), R01EY031209 (DS, CC, ML). Collection of human donor eyes for bulk RNA-sequencing studies was funded by the Arnold and Mabel Beckman Initiative for Macular Research (DS, CC).

## Conflict of Interest

CC is a consultant for Apellis.

The remaining authors declare that the research was conducted in the absence of any commercial or financial relationships that could be constructed as a potential conflict of interest.

## Publisher’s Note

All claims expressed in this article are solely those of the authors and do not necessarily represent those of their affiliated organizations, or those of the publisher, the editors and the reviewers. Any product that may be evaluated in this article, or claim that may be made by its manufacturer, is not guaranteed or endorsed by the publisher.
