# Supplementary material for: As in Real Estate, Location Matters: Cellular Expression of Complement Varies Between Macular and Peripheral Regions of the Retina and Supporting Tissues
Source: Front Immunol. 2022 Jun 15;13:895519. doi: 10.3389/fimmu.2022.895519 (PMC9240314; doi:10.3389/fimmu.2022.895519)
Supplement: Supplementary file 3 [file DataSheet_3.docx]

Supplementary Materials

**This file includes:**

Fig. S1

Tables S1 to S3

**Other Supplementary Materials for this manuscript include the following:**

Data S1 to S2


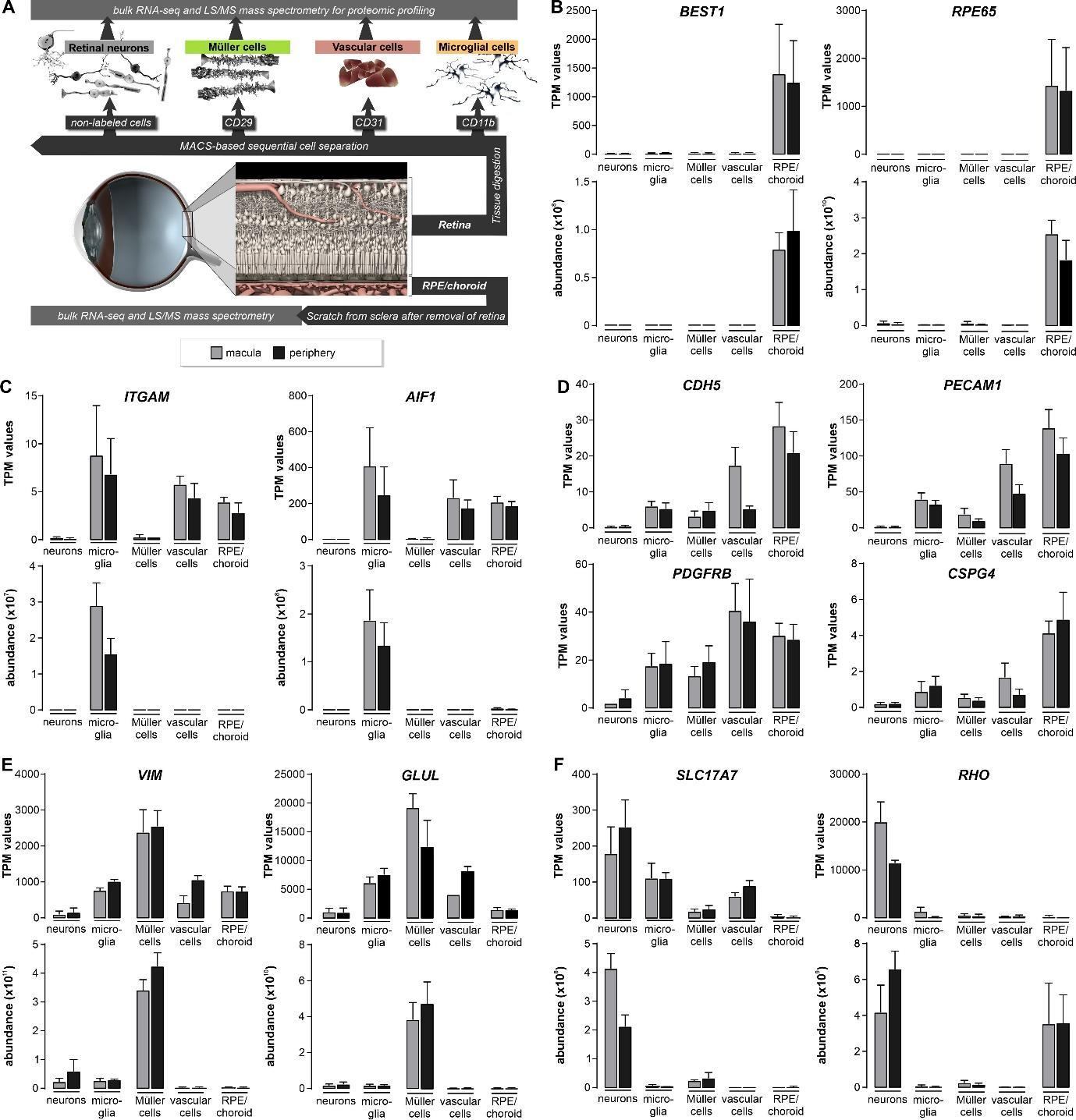
 Fig. S1.

Validation of enrichment of retinal cell types from macular (light grey) and peripheral (dark grey) tissue punches from human donor eyes using magnetic activated cell (MAC) sorting. Cell type-specific marker gene expression was confirmed by RNA-sequencing (TPM value) and protein profiling via quantitative LC MS/MS mass spectrometry (abundance).

A Scheme of the sequential MAC sorting procedure after retinal dissection and dissociation from human donor eyes. This includes the sequential purification of ITGAM (alias CD11B)-positive microglia/macrophages, CD31-positive vascular cells (pericytes, endothelial cells), CD29-positive macroglia (more than 85% Müller cells, few astrocytes) and the ITGAM (CD11B)/CD31/CD29-depleted flow through that is mainly comprised of the remaining retinal neurons.

B RPE/choroid samples were collected by scratching the tissue from the underlying scleral tissue punch after careful removal of the neuroretina. Bestrophin 1 (*BEST1*) and retinoid isomerohydrolase RPE65 (*RPE65*) were detected as marker genes for specific RPE enrichment.

C Integrin subunit alpha M (*ITGAM* alias *CD11B*) and allograft inflammatory factor 1 (*AIF1* alias *IBA1*) were analyzed to demonstrate their specifically high expression level in CD11B-positive cells. Note a slight contamination of the vascular cell fraction and the RPE/choroid. Since ITGAM and AIF1 is also expressed by macrophages, the relatively high expression of these two marker genes in the RPE/choroid is likely due to macrophages residing in the choroid.

D Cadherin 5 (*CDH5*) and platelet/endothelial cell adhesion molecule 1 (*PECAM1* alias *CD31*) were chosen to demonstrate enrichment of endothelial cells and platelet derived growth factor receptor beta (*PDGFRB*) and chondroitin sulfate proteoglycan 4 (*CSPG4* alias NG2) as marker for pericytes. Since the protein yield of CD31-positive cell population is very low, quantitative LC MS/MS mass spectrometry results are hard to evaluate and for that reason not included in this figure panel.

E To characterize the macroglial CD29-positive population, the classical Müller glia marker genes vimentin (*VIM*) and glutamine synthetase (*GLUL*) were chosen.

F The neuronal cell population (after depletion of microglia, vascular cells, Müller cells) is comprised by multiple neuronal subtypes. Rod photoreceptors should be one of the most abundant cell type and which is confirmed by clear enrichment of rhodopsin (*RHO*) in this cell population. Note the rhodopsin protein detection in RPE/choroid samples reflecting photoreceptor outer segment phagocytosis by RPE cells. In addition, we tested for enrichment of solute carrier family 17 member 7 (*SLC17A7* alias VGLUT1) in the neuronal population as known synaptic marker present in synaptic terminals of most neuronal cell types of the retina.

**Table S1**

**Overview of human donor tissue and metrics of samples used in the present study.**

| **Sample** | **Age/Sex** | **Phenotype** | **Tissue** | **Region** | **#Cells** | **Post mortem time** |
| --- | --- | --- | --- | --- | --- | --- |
| ***scRNA seq*** | | | | | |  |
| 20-1166-W | 84/F | Early AMD | Retina, Choroid | Macula, Periphery | 42051 | <6 h |
| 20-1438-W | 58/M | Normal | Retina, Choroid | Macula, Periphery | 31941 | <6 h |
| 20-1484-W | 89/M | Early AMD | Retina, Choroid | Macula, Periphery | 30784 | 8 h |
| 18-1077-W | 78/M | Normal | Retina | Macula, Periphery | 36909 | <6 h |
| 18-1132-P | 90/M | Normal | Retina | Macula, Periphery | 55476 | <6 h |
| ***Bulk RNA seq*** | | | | | |  |
| 11-1732-W | 69/M | Early AMD | Retina, RPE/Choroid | Macula, Periphery |  | <6 h |
| 11-2033-W | 85/F | Early AMD | Retina, RPE/Choroid | Macula, Periphery |  | <6 h |
| 11-2104-W | 95/M | Geo AMD | Retina, RPE/Choroid | Macula, Periphery |  | <6 h |
| 11-2253-W | 83/F | Early AMD | Retina, RPE/Choroid | Macula, Periphery |  | <6 h |
| 12-0094-W | 86/F | Geo AMD | Retina, RPE/Choroid | Macula, Periphery |  | <6 h |
| 12-0884-W | 86/F | Early AMD | Retina, RPE/Choroid | Macula, Periphery |  | <6 h |
| 12-0046-W | 85/M | Normal | Retina, RPE/Choroid | Periphery |  | <6 h |
| 12-0210-W | 84/M | Normal | Retina, RPE/Choroid | Periphery |  | <6 h |
| 12-0276-W | 92/F | Normal | Retina, RPE/Choroid | Macula, Periphery |  | <6 h |
| 12-0396-W | 86/F | Normal | Retina, RPE/Choroid | Macula, Periphery |  | <6 h |
| 12-0408-W | 83/M | Normal | Retina, RPE/Choroid | Macula, Periphery |  | <6 h |
| 12-0530-W | 83/M | Normal | Retina, RPE/Choroid | Macula, Periphery |  | <6 h |
| 12-0538-W | 84/M | Normal | Retina, RPE/Choroid | Macula, Periphery |  | <6 h |
| 12-0732-W | 83/F | Normal | Retina, RPE/Choroid | Macula, Periphery |  | <6 h |
| ***Bulk RNA seq of purified retinal cell populations*** | | | | | |  |
| 16-0928-OS | 76/M | Normal | MMVNRC | Macula, Periphery |  | <6 h |
| 16-0928-OD | 76/M | Normal | MMVNRC | Macula, Periphery |  | <6 h |
| ***Mass spectrometric proteome profiling of purified retinal cell populations*** | | | | | |  |
| 19-0012-OS | 78/M | Normal | MMVNRC | Macula, Periphery |  | 26 h |
| 19-0013-OS | 89/M | Normal | MMVNRC | Macula, Periphery |  | 30 h |
| 19-0013-OD | 89/M | Normal | MMVNRC | Macula, Periphery |  | 30 h |
| 19-0015-OS | 59/F | Normal | MMVNRC | Macula, Periphery |  | 26 h |
| 19-0015-OS | 78/M | Normal | MMVNRC | Macula, Periphery |  | 23 h |
|  |  |  |  |  |  |  |
| OS…os sinister | |  |  |  |  |  |
| OD…od dexter | |  |  |  |  |  |
| MMVNRC=Müller cells, microglia, vascular cells, neurons, RPE/choroid | | | |  |  |  |

**Table S2.**

**Primary and secondary antibodies for Western Blot detection.**

| **Primary**  **antibody** | **Species** | **Company** | **Catalogue number/**  **Reference** | **Dilution/Concentration** |
| --- | --- | --- | --- | --- |
| anti-C1s | rabbit | Proteintech (Rosemont, IL, USA) | #14554‐1‐AP | 1:1000 |
| anti-C3 | rabbit | Abcam (Cambridge, MA, USA) | #ab181147 | 1:1000 |
| anti-C7 | goat | Tecomedical (Sissach, Switzerland) | A308 | 1:250 |
| anti-FI | goat | Quidel (San Diego,  CA, USA) | A313/#181137 | 1:200 |
| anti-FH | goat | Merck (Burlington, MA, USA) | #341276 | 1:250 |

| **Secondary antibody** |
| --- |

| anti‐  rabbit Ig‐  HRP | goat | Dianova (Hamburg, Germany) | #111‐035‐003 | 1:10000 |
| --- | --- | --- | --- | --- |
| anti-goat Ig-HRP | rabbit | Dianova (Hamburg, Germany) | #305‐035‐003 | 1:10000 |

| anti-goat | donkey | Dianova (Hamburg, Germany) | 705-165-147 | 1:500 |
| --- | --- | --- | --- | --- |
| anti-rabbit | donkey | Invitrogen | A-21206 | 1:500 |
| anti-goat | donkey | Dianova (Hamburg, Germany) | 705-225-147 | 1:500 |

**Table S3.**

**Sera and recombinant proteins.**

| **Recombinant Proteins** | **Company** | **Catalogue number/**  **Reference** |
| --- | --- | --- |
| Human complement protein C1s | Merck KGaA (Darmstadt, Germany) | #204879 |
| Human complement protein C3 | CompTech (Tyler, Texas, USA) | A113 |
| Human complement protein C7 | CompTech (Tyler, Texas, USA) | A124 |
| Human complement protein FI | CompTech (Tyler, Texas, USA) | A138 |
| Human complement protein FH | CompTech (Tyler, Texas, USA) | A137 |
| **Sera** |  |  |
| Normal human serum | CompTech (Tyler, Texas, USA) | NHS |
| C1s-depleted human serum | CompTech (Tyler, Texas, USA) | A304 |
| C3-depleted human serum | CompTech (Tyler, Texas, USA) | A314 |
| C7-depleted human serum | CompTech (Tyler, Texas, USA) | A324 |
| CFI-depleted human serum | CompTech (Tyler, Texas, USA) | A338 |
| CFH-depleted human serum | CompTech (Tyler, Texas, USA) | A337 |

Data S1. (separate file)

Significant complement gene expression assessed by scRNA-seq for retinal and choroidal cell types in early AMD and normal tissue comparing macula and periphery.

Data S2. (separate file)

Significant complement gene expression assessed by bulk RNA-seq for whole retina and RPE/choroid/scleral samples in early/late AMD and normal tissue comparing macula and periphery.
